# Unveiling the power of *Pueraria lobata*: a comprehensive exploration of its medicinal and edible potentials

**DOI:** 10.3389/fphar.2025.1578472

**Published:** 2025-07-10

**Authors:** Baixin Kou, Lingkun Meng, Mengya Zhao, Haidong Wang, Chunyang Lu, Mingming Yan, Guangzhe Li

**Affiliations:** ^1^ School of Pharmaceutical Sciences, Changchun University of Chinese Medicine, Changchun, China; ^2^ Jilin Ginseng Academy, Changchun University of Chinese Medicine, Changchun, China; ^3^ Jilin Provincial Science and Technology Innovation Center of Health Food of Chinese Medicine, Changchun University of Chinese Medicine, Changchun, China

**Keywords:** *Pueraria lobata* (Willd.) Ohwi, herbal textual research, phytochemistry, pharmacology, edibility, puerarin

## Abstract

**Background:**

*Pueraria lobata* (Willd.) Ohwi, a perennial vine native to China, has significant medicinal and edible value. Its roots are used as medicine and are known as kudzu (Chinese: *Gegen*) and were first recorded in *Shen Nong Ben Cao Jing*. In addition, its roots and powder can be made into food.

**Aim of the study:**

To integrate information on the source of *Pueraria lobata* (PL), summarize the evolution of its medicinal and edible value, and generalize its chemical composition, biosynthetic pathways, metabolism, and biological activity.

**Materials and Methods:**

A comprehensive literature search (1975–2025) was conducted across ScienceDirect, Google Scholar, PubMed, Web of Science, CNKI, WFO (www.worldfloraonline.org), MPNS (https://mpsn.kew.org), Changchun University of Chinese Medicine Library collections, and SciFinder. Ancient applications were validated through analysis of classic Chinese medical literature.

**Results:**

PL is predominantly found in Asia, Europe, and America, with significant populations in China’s Zhejiang, Jiangxi, Jiangsu, Guangdong, and Guangxi provinces. The plant comprises flavonoids, triterpenoids, and coumarins, including isoflavonoids like daidzein and puerarin, which are synthesized via diverse pathways. Metabolites produced from liver or intestinal reactions are crucial to PL’s effectiveness. Key components include puerarin, daidzein, genistein, biochanin A, and formononetin. In China, PL is incredibly versatile, being used in a wide range of foods, teas, preservatives, dairy products, *etc.*

**Conclusion:**

Its extensive biological activities benefit the human body, with particular emphasis on liver protection, anti-osteoporosis, and anti-diabetic effects. These attributes highlight the potential for developing health foods, revealing PL’s promising prospects in the pharmaceuticals and nutritional healthcare industries.

## 1 Introduction

*Pueraria lobata* (Willd.) Ohwi is a perennial vine native to China, mostly found in Asia, and later introduced to the Americas and Europe, that contains micromolecules such as flavonoids, triterpenoids, and coumarins, as well as macromolecules such as polysaccharides and proteins (Gao et al., 2024). PL has a wide range of biological activities, such as lowering blood pressure, lowering sugar, detoxification, anti-virus, anti-osteoporosis, anti-inflammatory, antioxidant, and anti-aging (Zhou et al., 2014), which makes it an unparalleled delicacy. It has been used for thousands of years, as early as the *Shen Nong Ben Cao Jing*; it is categorized as a middle grade. *Ben Cao Jing Ji Zhu* recorded its edible nature, and it has been used until now.

Although PL has been used in China for thousands of years, the records of PL in successive *Materia Medica* may be somewhat confusing due to climatic changes and dynastic changes. In order to clarify the origin and improve the quality of PL, it is important to verify the name, place of origin, plant morphology, and records of medicinal use and consumption of PL in ancient times. With the current demand for a healthier state of affairs, herbs such as PL, which can be used both medicinally and as a food, have gradually gained popularity, and the number of related studies has been increasing in recent years. In addition, at this stage, the research on the synthesis pathway and metabolic pathway of isoflavones of kudzu lacks generalization and summarization, and the medicinal values and food functions of PL are not comprehensively expressed, which seriously affects its development. The present review is intended to provide a comprehensive representation of PL in terms of its basal origin, phytochemical profile, synthesis, metabolism, bioactivities, and comprehensive utilization for food consumption and medicinal use.

## 2 Materials and methods

### 2.1 Search strategy

An online literature search was carried out at ScienceDirect, Google Scholar, PubMed, Web of Science, CNKI, WFO (https://www.worldfloraonline.org), MPNS (https://mpsn.kew.org), Changchun University of Chinese Medicine Library collections, and SciFinder, covering 1975 to 2025. The following keywords were used: “*Pueraria lobata*” and “phytochemistry” or “pharmacology” or “edibility.” The references of all retrieved articles were also reviewed to include relevant literature. Consistent selection criteria were applied across all databases, with duplicate studies eliminated through a two-step process: automated detection using Zotero software, followed by manual cross-checking. Ancient applications were validated through analysis of classic Chinese medical literature. Comprehensive searches encompassed medicinal and dietary records containing the following nomenclature: “*Ge Gen*,” “*Ge*,” “*Lu Huo*,” “*Huang Jin*,” “Ye *Ge*,” and “*Gan Ge*.” Relevant prescriptions were compiled following duplicate removal via cross-database verification, with remaining materials organized chronologically based on textual origins.

### 2.2 Selection criteria

The inclusion criteria are as follows: PL, PL extracts, and its compounds are the subjects of the study; the diseases and physiological processes targeted by PL; the study design is clear, and the study results involve the exploration of relevant mechanisms; the latest research on PL’s clinical applications and formulations is included; the literature was published within the past 50 years, unless it holds significant historical value; the research must explicitly describe molecular mechanisms or signaling pathways and their effects on bioavailability or efficacy.

Exclusion criteria comprised: 1) literature reviews lacking clear research parameters (subjects/methods/mechanisms), 2) methodologically deficient studies (small sample sizes, unreliable results, and low quality), 3) duplicate publications, 4) non-data-driven articles (conference abstracts, editorials, and opinion pieces), and 5) non-peer-reviewed/unpublished research. Standardized extraction protocols ensured data uniformity, with dual independent review by researchers enhancing analytical rigor.

## 3 Herbal textual research

### 3.1 Name

The name of *Ge Gen* was first mentioned in *Shen Nong Ben Cao Jing* and recorded as the proper name of this product. Other herbal works, such as *Xin Xiu Ben Cao*, *Zheng Lei Ben Cao*, *Ben Cao Gang Mu*, and *Ben Cao Cong Xin*, in the record of this product, use *Ge Gen* as the proper name, which has been used until now. The synonyms are *Ji Qi Gen* (*Shen Nong Ben Cao Jing* and *Ben Cao Jing Ji Zhu*), *Lu Huo* (*Ben Cao Jing Ji Zhu* and *Ming Yi Bie Lu*), Huang Jin (*Ben Cao Jing Ji Zhu* and *Ming Yi Bie Lu*), *Ge Dou* (*Ben Cao Pin Hui Jing Yao*), and *Gan Ge* (*Pao Zhi Quan Shu*).

### 3.2 Place

There are different opinions about the origin of PL. *Shen Nong Ben Cao Jing* records that it “grows in mountain valleys,” and *Ben Cao Tu Jing* states that it is especially abundant in Jiangsu and Zhejiang provinces. “The essence of the herb” pointed to Jiangsu, Zhejiang, and Jiangxi provinces as the road to the production area. The name of the plant in the real map test recorded PL as a cultivated plant; there are also wild versions. Nowadays, there is much PL in Guangdong, Guangxi, Hainan, Zhejiang, and Jiangsu provinces, and wild PL is found in Hebei, Henan, Jiangxi, Hubei, and Hunan provinces (Table 1). Therefore, PL-producing areas are Zhejiang, Jiangxi, Jiangsu, Guangdong, Guangxi, Hainan, Hebei, Henan, Hubei, and Hunan provinces. With the change of climate, PL is now mainly grown commercially in Hunan, Zhejiang, Jiangxi, and Hubei provinces.

**TABLE 1:** Names, characteristics, and places of *Pueraria lobata* (Willd.) Ohwi in *Materia Medica* throughout the ages.

Dynasty	Reference	Name	Characteristic	Place	Origin
Western Han	*Shen Nong Ben Cao Jing*	*Ge Gen* and *Ji Qi Gen*	—	—	*P. lobata*
Liang	*Ben Cao Jing Ji Zhu*	*Ji Qi Gen*, *Lu Huo*, and *Huang Jin*	—	*Jiangxi*	*P. thomsonii*
Liang	*Ming Yi Bie Lu*	*Ge Gen*, *Lu Huo*, and *Huang Jin*	—	*Sichuan*	*P. thomsonii*
Tang	*Shi Liao Ben Cao*	*Ge Gen*	—	—	*P. thomsonii*
Song	*Qing Yi Lu*	*Zou Gen Mei*	—	—	*P. lobata*
Song	*Ben Cao Tu Jing*	*Ge Gen*	PL grows seedlings in the spring, the purple vine is 3.168–6.336 m long, the green leaves are similar to those of the seized, the purple flowers like pea blossoms are pollinated in July, and the purple-black root is shaped like an arm, and the root is harvested at noon of the fifth day of the fifth lunar month, and dried in the sun	*Jiangsu* and *Zhejiang*	*P. lobata*
Song	*Ben Cao Yan Yi*	*Ge Gen*	—	*Jiangxi*	*P. thomsonii*
Ming	*Ben Cao Pin Hui Jing Yao*	*Ji Qi Gen*, *Lu Huo*, *Huang Jin*, and *Ge Dou*	—	*Jiangsu*, *Zhejiang*, and *Jiangxi*	*P. lobata*
Ming	*Ben Cao Meng Quan*	*Ge Gen*	Its vines grow long and swirling	*Gansu* and *Jiangsu*	*P. lobata/P. thomsonii*
Ming	*Ben Cao Gang Mu*	*Ge Gen*	The roots of this plant are purple on the outside and white on the inside and can be seven or eight feet long. The leaves have three tips, like a maple leaf but longer, and are green on the front and light in color on the back. The flowers are in bunches, reddish purple, and very pretty. The pods look like small yellow bean pods and are also hairy. The seeds inside are green and flat, like the kernel of a salt plum, and have a fishy flavor when eaten raw, and are picked in August and September	—	*P. lobata*
Ming	*Ben Cao Yi Du*	*Ge Gen*	The roots of this plant are purple on the outside and white on the inside, and are trailing. The leaves have three tips and are green on the front and pale on the back. The flowers are bunches of reddish purple. The pods look like small yellow bean pods	—	*P. lobata*
Qing	*Ben Cao Cheng Ya Pu Jie*	*Ge Gen*	Spring seedlings, vines can grow two or three feet, leaves three-pointed like long maple leaves, verdant color. July blossom, a bunch of purple and pink, like pea flowers, fruit like small soybean pods, pods have hair, inside the seed green flat, like salt plum kernel, eaten raw has a fishy taste, this is the kudzu. Its root is as thick as an arm, the outer skin purple and black inside white, can be made into powder to eat. The flowers are reddish purple, the fruit is like a yellow pod, the seed is like a plum kernel, and it has a fishy flavor when eaten raw	*Fujian*, *Guangdong*, *Jiangsu*, and *Zhejiang*	*P. lobata*
Qing	*Ben Cao Chong Yuan*	*Ge Gen*	Spring seedlings, vines spreading, roots like an arm thick, purple and black skin inside white. The flowers are reddish purple, the fruits are like yellow pods, and the seeds inside are like prune kernels, which have a fishy flavor when eaten raw	*Jiangsu* and *Zhejiang*	*P. lobata*
Qing	*Pao Zhi Quan Shu*	*Gan Ge*	—	—	*P. lobata*
Qing	*Pao Zhi Da Fa*	*Ge Gen*	—	—	*P. thomsonii*
Qing	*Ben Cao Xiang Jie*	*Ge Gen*	Vines 3.168–6.336 m long, roots purple on the outside, white on the inside	—	*P. lobata*

### 3.3 Origin

**FIGURE 1:**
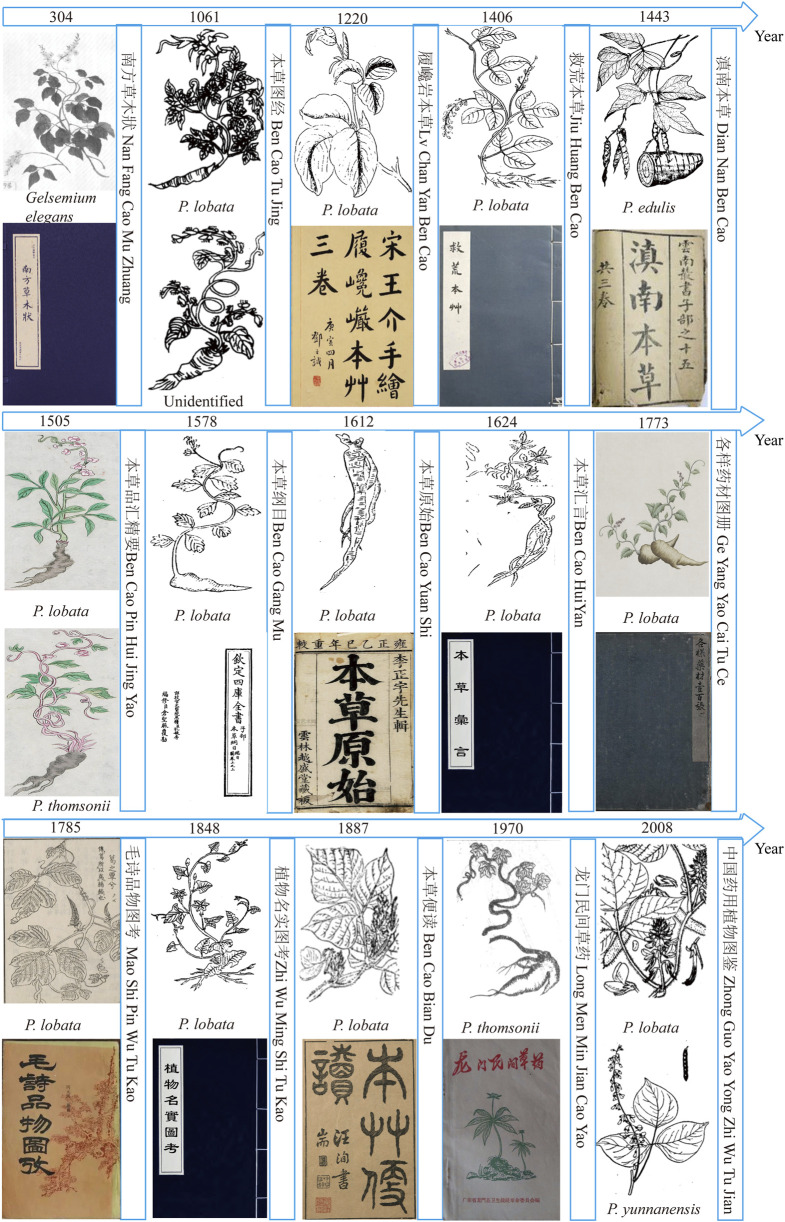
Medicinal plant illustrations of Ge in the *Materia Medica*.

After integrating the herbs of the past dynasties, Figure 1 was obtained. Before the Song Dynasty, the basal origin was not clearly defined, and there was often a mixed use; after the Song Dynasty, both *P. lobata* and *Pueraria thomsonii* were used as the source of kudzu. From the Ming and Qing dynasties to modern times, *P. lobata* was mainly used as a medicine and was categorized as the authentic source of *P. lobata* in the Chinese Pharmacopoeia.

#### 3.3.1 Song and pre-Song dynasties

Before the Song Dynasty, there was no clear description of the plant of PL. *Nan Fang Cao Mu Zhuang* clearly records that “*Ye Ge* is a kind of *Duan Chang Cao*, which is called *Huo Ba Hua* by people in southern Yunnan province.” Combined with the historical background and the depiction in the picture, based on the available literature and speculation, what is depicted in this book may be *Gelsemium elegans*, which is also known as *Gou Wen.* It is highly poisonous, and *G. elegans* is not a source of medicinal plants. It suggests that there may have been cases of PL with the same name before the Han Dynasty. The form of PL can be traced back to the Northern Song Dynasty. The *Ben Cao Tu Jing* contains: “PL grows seedlings in the spring, the purple vine is 3.168–6.336 m long, the green leaves are similar to those of the seized, the purple flowers like pea blossoms are pollinated in July, and the purple-black root is shaped like an arm, and the root is harvested at noon of the fifth day of the fifth lunar month and dried in the sun.” This is the first description of the morphology of the PL, with illustrations of *Haizhou* (Jiangsu province) kudzu and *Chengzhou* (Gansu province) kudzu, the latter of which has a single leaf and is clearly not a *Pueraria* plant, whereas the *Haizhou* kudzu has three leaflets, pods, and may be *P. lobata*. From the above, it can be seen that during the Song Dynasty, different varieties of plants were already used as kudzu mixes. The Northern Song Dynasty’s *Ben Cao Yan Yi* recorded that: “During the winter, fresh PL is kneaded in water to produce starch, which settles and is made into starch cubes. Boil water first, then break the starch block into small pieces and put it in to boil, until the color is like glue and it becomes very tough. Fish it out and mix it in honey water to eat; add some grated ginger for better flavor. Some people also add these starch pieces to the tea to cook to entertain guests, but just sweet, nothing special benefits.” The use of *P. thomsonii* is clearly reflected here, and it can be assumed that after the Song Dynasty, the species of kudzu gradually diversified, with both *P. lobata* and *P. thomsonii* being used.

#### 3.3.2 Ming and Qing dynasties

By the Ming Dynasty, the phytomorphological drawings of PL contained in *Jiu Huang Ben Cao*, and other herbaceous texts were all likely to be *P. lobata*, but *P. thomsonii* had been recognized as having a less medicinal, but rather powdery and edible character, but its phytomorphology had not been described. It is stated that “the sweet-tasting kudzu is sweet kudzu, and the bitter-tasting kudzu is bitter kudzu,” and the sweet kudzu it describes is probably the edible kudzu, *Pueraria edulis*, which is clearly distinguished from the other kudzu plants by the distinctly terete tuberous roots and the banded, rectangular-shaped pods in *Dian Nan Ben Cao*. Alternatively, bitter kudzu may be *Pueraria peduncularis*, also known as Yunnan Geteng, which is not used medicinally. *Ben Cao Pin Hui Jing Yao* redrew the *Haizhou* kudzu and *Chengzhou* kudzu from the *Ben Cao Tu Jing*, adding color to the previous black-and-white illustration, and it is clear that both are kudzu, with *Haizhou* kudzu morphologically characterized by a trilobed terminal leaflet, occasionally entire, presumed to be PL. The morphology of *Chengzhou* kudzu is characterized by trilobed terminal leaflets, occasionally entire, and a large degree of tuberous expansion, presumably *P. thomsonii*.

In more recent times, the morphology of *P. thomsonii* has been more carefully drawn in the *Long Men Min Jian Cao Yao*, and the expanded state of its tuberous roots has generally become an identifying feature. Therefore, the Chinese Pharmacopoeia specifies the authentic source of PL (China, 2020), which reinforces the fact that PL has been used as a medicine for thousands of years.

### 3.4 Usage

#### 3.4.1 Medicinal use

The use of PL was first described in the *Shen Nong Ben Cao Jing*. It is mainly used for treating thirst, fever, vomiting, and various kinds of paralysis. Since then, the medicinal effects of PL have been documented throughout the ages, including relieving pain, sweating, penetrating rashes, and relieving depression. *Xin Xiu Ben Cao* recorded that “its flowers and small bean flowers dried powder, take a little; when poor drinking, that is, the flower of PL for a good antidote to alcohol.” The efficacy of raw and cooked products is distinguished in the *Ben Cao Meng Quan*: raw can abort fetuses, steamed and cooked to remove the poison of wine (Table 2). In summary, PL is sweet, pungent, cool, and non-toxic, with medicinal values of relieving muscle fever, generating fluids to quench thirst, penetrating rashes, raising the sun to stop diarrhea, invigorating the meridians, and detoxifying alcohol, which is in line with the Chinese Pharmacopoeia (China, 2020).

**TABLE 2:** Main efficiency of *Pueraria lobata* (Willd.) Ohwi in books throughout the ages.

Dynasty	Reference	Main treatment
Western Han	*Shen Nong Ben Cao Jing*	Treating thirst, quenching vomiting, detoxification, and paralysis
Liang	*Ben Cao Jing Ji Zhu*	Pain relief, and sweating
Tang	*Xin Xiu Ben Cao*	Raw juice to reduce fever and relieve alcoholism
Song	*Ben Cao Tu Jing*	Raw juice stops pain; leaves stop bleeding
Song	*Zheng Lei Ben Cao*	Stopping vomiting, detoxifying alcohol, and relieving fever
Song	*Ben Cao Yan Yi*	Diuretic and alcohol detoxification
Ming	*Ben Cao Meng Quan*	Promoting the production of body fluid and quenching thirst, quenching the poison of alcohol, relieving pain, vomiting, and relieving fever
Ming	*Ben Cao Gang Mu*	Relieve fever and vomiting
Ming	*Ben Cao Cheng Ya Pu Jie*	Anti-toxic and stop paralysis
Ming	*Ben Cao Zheng Yao*	Anti-nausea, anti-diarrhea, and detoxification
Ming	*Ben Cao Tong Xuan*	Relieves pain, quenches thirst, and penetrates rashes
Qing	*Ben Cao Yi Du*	Reducing fever, relieving vomiting, stopping diarrhea, relieving asthma, relieving alcoholism, and dispersing depression

#### 3.4.2 Edible use

Ancient texts on the use of PL as a food application can be traced as far back as the Northern and Southern Dynasties (220–589), and *Ben Cao Jing Ji Zhu* clearly states that kudzus are steamed and eaten by people. The earliest application of kudzu powder can be traced back to the Tang Dynasty (713–741), *Shi Liao Ben Cao* recorded that: raw kudzu is harvested in winter, kneaded out of the powder with water, made into pallets, fried in boiling soup, removed, and put into the soup liquid, waiting for its color like glue, with white and toughness. Mixed with honey and eaten, crushed ginger is also good to treat fever, drunkenness, and thirst; it can facilitate urination and is also refreshing; when cut and eaten as a tea, it is very sweet, and raw kudzu directly simmered has a nourishing effect. In 978, *Tai Ping Sheng Hui Fang* was published, in which are recorded the *Ge Fen Suo Bing Fang*, *Ge Fen Zhou Fang*, *Fa Han Chi Zhou Fang*, *Ge Fen Ba Dao Fang*, and *Fen Zhou Fang*. Thereafter, during the Song and Yuan dynasties, PL was consumed mainly for the treatment of thirst and stroke in old age. In the Ming Dynasty, the therapeutic applications of PL were more extensive, including relieving cough, resolving phlegm, quenching thirst, filling hunger, and quenching summer heat, *etc.*, and there were even external prescriptions for moisturizing and perfuming the skin and body, such as the *Fu Shen Xiang Ti Fang* (Table 3).

**TABLE 3:** Therapeutic recipes related to *Pueraria lobata* (Willd.) Ohwi in books throughout the ages.

Dynasty	Reference/	Prescription	Treatment	Formula	Preparation/method of administration
Song	*Tai Ping Sheng Hui Fang*	*Ge Fen Suo Bing Fang*	Stroke with heat in the heart and spleen	*Pueraria* powder (238 g), *Schizonepeta tenuifolia* (4.5 g), and Sojae Semen Praeparatum (30 g)	Decoct edamame and thorns in 900 mL of water, and take 750 mL, remove the dregs, and add kudzu powder as juice, cook, and eat on an empty stomach
Song	*Tai Ping Sheng Hui Fang*	*Ge Fen Zhou Fang*	Suffer a paralyzing stroke	White sorghum rice (300 g) and *Pueraria* powder (238 g)	Mix white bean rice and kudzu powder together and cook in light bean drum juice
Song	*Tai Ping Sheng Hui Fang*	*Fa Han Chi Zhou Fang*	Suffer a paralyzing stroke	Sojae Semen Praeparatum (15 g), *Schizonepeta tenuifolia* (4.5 g), *Ephedrae herba* (1.785 g), *Pueraria lobata* (59.5 g), *Gardenia jasminoides* Ellis (1.785 g), *Gypsum fibrosum* (178.5 g), Bulbus Allii Fistulosi (10 g), *Zingiber officinale Roscoe* (29.75 g), and *Oryza sativa* L. (30 g)	Decoct 900 mL of water to 400 mL, discard the dregs of the medicine, add round-grained rice to make a thin porridge, and consume
Song	*Tai Ping Sheng Hui Fang*	*Ge Fen Ba Dao Fang*	Suffer a paralyzing stroke	*Pueraria* powder (476 g), *Schizonepeta tenuifolia* (59.5 g), Bulbus Allii Fistulosi (30 g), *Zingiber officinale Roscoe* (29.75 g), *Zanthoxylum schinifolium* (15 g), Sojae Semen Praeparatum (15 g), Salt (29.75 g), and bone marrow of sheep’s testicles (59.5 g)	In 1,500 mL of water, *Schizonepeta tenuifolia* is first decocted, and 600 mL of juice is extracted and mixed with *Pueraria* powder, boiled, and consumed
Song	*Tai Ping Sheng Hui Fang*	*Fen Zhou Fang*	Burning hot in the chest	*Pueraria* powder (476 g) and chestnut rice (477.5 g)	Soak the chestnut rice in water overnight, and the next day, take the corn and *Pueraria* powder that have been soaked in the water and cook them into a porridge
Song	*Tai Ping Sheng Hui Fang*	*—*	Dysentery characterized by blood	*Pueraria* powder (238 g) and honey (15–30 g)	Boil in 500 mL of water, stir and mix well, and take in two divided doses on an empty stomach
Song	*Yang Lao Feng Qin Shu*	*Shi Zhi Lao Ren Zhong Feng Fang*	Geriatric stroke	*Pueraria* powder (297.5 g), *Schizonepeta tenuifolia* (4.5 g), and Sojae Semen Praeparatum (75 g)	After decocting the two herbs, take out the juice, add a little of the onion, pepper, seasoning, and bouillon, and take it on an empty stomach
Song	*Sheng Ji Fang*	*Ge Fen Fan Fang*	Middle migraine and thirst	*Pueraria lobata* (238 g) and maize rice (300 g)	Soak rice in syrupy water, strain it out, mix it with *Pueraria* powder, cook it in Sojae Semen Praeparatum juice over a rapid fire, and eat it with five flavors of onion and white onion three times a day, and still do not eat anything
Song	*Shi Zhai Bai Yi Xuan Fang*	*Shui Hu Lu Wan*	Starve	Chuan Bai Yao (178.5 g), *Panax ginseng* (5.95 g), *Glycyrrhizae radix et rhizoma* (29.75 g), *Ophiopogon japonicus* (29.75 g), *Prunus mume* (29.75 g), Lignesse charcoal (29.75 g), and *Pueraria lobata* (29.75 g)	Add all ingredients into a fine powder and make pills as big as a chicken head with flour paste. Take one pill each time and dissolve it in the mouth. When traveling in the summer months, one pill can last for 1 day
Song	*Shi Zhai Bai Yi Xuan Fang*	*Shou Wei San*	Have diarrhea and vomiting	Mean Score *Panax ginseng*, *Atractylodes macrocephala, Smilax china, Dioscoreae rhizoma, Dolichos lablab, Pueraria lobata, Arisaema erubescens, Glycyrrhizae radix et rhizoma, Agastache rugosa, Saposhnikovia divaricata*, and *Gastrodia elata Bl*	One cent per serving. Water, 20 seeds of winter melon, and a small piece of ginger. Decoct to four parts. Take warm.
Yuan	*Yin Shan Zheng Yao*	*Ge Fen Geng*	Stroke, wind-heat in the heart and spleen	*Pueraria* powder (238 g); *Schizonepeta tenuifolia* (59.5 g), and Sojae Semen Praeparatum (45 g)	First boil *Schizonepeta tenuifolia* and Sojae Semen Praeparatum in water to 60°C–70°C and remove the dregs. *Pueraria* powder is used as a dough, cooked in the middle of the juice, and taken on an empty stomach
Yuan	*Yin Shan Zheng Yao*	*Ju Pi Xing Cheng Tang*	Inebriated and unable to understand	Orange peel (955 g), tangerine peel (955 g), sandalwood (238 g), *Pueraria* flowers (477.5 g), mung bean (477.5 g), ginseng (119 g), white cardamom (119 g), and stir-fried salt (357 g)	The above is finely powdered
Ming	*Jiu Huang Ben Cao*	*Zha Ge Hua*	Starve	*Pueraria* flowers	Can also be dried and fried
Ming	*Zhu Yu Shan Fang Za Bu*	*Yan Zheng Ge*	Starve	*Pueraria* powder	*Pueraria* powder is boiled and steamed with a little sugar, rice dosa, or pork cubes made into patties
Ming	*Zhu Yu Shan Fang Za Bu*	*Fa Zhi Sheng Jiang*	Nausea and do not want to eat	Ginger (595 g), cinnamon (59.5 g), green peel (59.5 g), tangerine peel (59.5 g), pinellia (59.5 g), atractylodes (59.5 g), tomato (148.75 g), clove (148.75 g), fragrance (148.75 g), white bean kernel (89.25 g), white poria cocos (89.25 g), amomum (89.25 g), *Pueraria lobata* (29.75 g), and licorice (29.75 g)	Take as much as you like
Ming	*Yin Zhuan Fu Shi Jian*	*Fu Shen Xiang Ti Fang*	Fragrance moisturizing	*Pueraria* powder (955 g), green woodruff (119 g), ephedra root (119 g), fried sweetgum (119 g), licorice (119 g), and patchouli (119 g)	The above herbs are crushed to a powder, mixed with *Pueraria* powder, put in a thin silken bag, and worn on the body after bathing
Ming	*Yin Zhuan Fu Shi Jian*	*Shi Shuang Qing Ge Bing Fang*	Regulating qi, resolving phlegm and relieving cough	Persimmon frost (2,292 g), orange peel (477.5 g), *Platycodon grandiflorus* (238 g), *Mentha piperita* (357 g), *Pueraria lobata* (119 g), Fenghuang (238 g), and ice tablet (5.95 g)	Grind the above herbs together and add licorice paste to make cakes for consumption
Ming	*Yin Zhuan Fu Shi Jian*	*Mei Su Wan Fang*	Eliminating summer heat by cooling	Ume flesh (119 g), *Pueraria lobata* (35.7 g), sandalwood (5.95 g), perilla leaves (17.85 g), fried salt (5.95 g), and sugar (955 g).	Take the above herbs and make a powder, grind the flesh of umeboshi into a paste, mixing the other powders, and use it as small pills
Ming	*Yin Zhuan Fu Shi Jian*	*Zui Xiang Bao Xie*	Sputum-thinning effect	*Pericarpium citri reticulatae* (238 g), Semen Armeniacae (23.8 g), soy bean (95.2 g), Herba miltiorrhizae (142.8 g), ginger (17.85 g), clove (5.95 g), *Pueraria lobata* (178.5 g), soya bean kernel (59.5 g), salt (59.5 g), and croton (7 g)	Boil the above herbs in 600 mL of water until the water dries up, remove the croton, and dry it in the sun. Chew it finely and take it in white soup
Ming	*Yin Zhuan Fu Shi Jian*	*Gan Lan Wan*	Relieving cough and sputum	Baiyao decoction (29.75 g), ume plum (47.6 g), papaya (11.9 g), *Pueraria lobata* (11.9 g), sandalwood (2.9 75 g), and *Glycyrrhiza uralensis* (29.75 g)	Make pills and dry them in the sun
Ming	*Yin Zhuan Fu Shi Jian*	*Zhi* Tan *Kuai Qi Xiao Ge Shi Shen Fang*	Eliminating phlegm and eliminating diaphragm	Pre-herb: *Pinellia ternata* (955 g), prepared Nan Xing (955 g), ginger (955 g), soap jerky (955 g), and alum (955 g). Posterior Herbs: Green peel (477.5 g), Chen Pei (477.5 g), stir-fried radish seed (477.5 g), stir-fried Su Zi (477.5 g), stir-fried Shen Zi (477.5 g), ginger parsley (477.5 g), stir-fried malt (477.5 g), *Pueraria lobata* (477.5 g), almond (477.5 g), and Yamcha (477.5 g)	The five flavors of the former medicine are boiled in water, to the heart of the southern star without white defense, and the soap horn does not need to be chopped ginger. With the South Star, half-summer dry or fire-roasted, with the latter medicine, and the former flavors and mixed with the former for fine powder for fine powder, soaked in ginger natural juice, steamed cake for the pills, each serving of 50 or 60 pills before going to bed. Serve with tea and wine
Ming	*Yin Zhuan Fu Shi Jian*	*Qian Li Bu Yin Shui Bu Ke Fang*	Quenching	White honey (71.4 g), licorice (59.5 g), peppermint (59.5 g), plum flesh (59.5 g), *Poria cocos* (208.25 g), *Pueraria lobata* (59.5 g), salt plum (59.5 g), and steamed *Polygonum multiflorum* (148.75 g)	Mix together and make a honey pill
Qing	*Yang Xiao Lu*	*Yu Lu Shuang*	Starve	Smallpox powder (238 g), *Pueraria lobata* (59.5 g), orange stem (59.5 g), bean powder (595 g), some dried mint, and sugar (476 g)	Stir up small poxpowder, dry kudzu, orange stalks and bean powder, wetting mint with water, let go of the collection of water traces, flat in the bottom of the basin, put the powder on the top; and then a layer of silk, and then add mint, cover, and seal, will be boiled through the soup, take out the cold, remove the next one or 2 days, add sugar 476 g, and evenly printed molds
Qing	*Zhou Pu*	*Ge Gen Zhou*	Remove thirst	—	—

Modern research has shown that PL has a rich nutritional value because it contains a large amount of starch, polyphenols, amino acids, *etc.* (Xu et al., 2022; Wang J. et al., 2023). Yang et al. (2022) showed that the apparent content of resistant starch of PL was 23.14%, the molecular weight was 1.93 × 10^7^ Da, and the solubility and solubility of starch of PL were 38.51% and 28.10 g/g, respectively. This suggests that PL could be a potential source of dietary fiber and that the starch of PL has a better application in food processing. Pang et al. (2023) found that the foaming capacity (200%) and foaming stability (97.5%) of PL’s protein increased with hydrolysis. This shows that hydrolysis contributes to the alteration of the functional properties of PL, offering the possibility of its food applicability. In addition, the leaf extract of PL contains amino acids such as arginine, valine, and proline, including 0.23% Na, 3.5% K, and 0.24% Ca (Kemertelidze et al., 2008). This reinforces the richness of PL’s prospects for food development.

Nutritional analysis revealed PL exhibits a distinct nutritional profile characterized by: 449.85 mg/g starch content, 3.81 g/100 g soluble dietary fiber, 10.23 g/100 g insoluble dietary fiber, and 106.24 g/kg crude protein content (Fu M., 2023). Compared to other products that provide dietary fiber (sweet potato 2.21%, yam 7.3%), PL is high in dietary fiber and low in calories, which provides a unique basis for its food value (Zhang Z. et al., 2023; Li Y. et al., 2023). Among them, *Puerariae lobatae* Radix-resistant starch is a potential novel prebiotic that is able to repair the intestinal barrier and reverse the pathology of the organism, with obvious health effects (Lian et al., 2023). Additionally, its homogeneous polysaccharide supports intestinal homeostasis by increasing short-chain fatty acid levels and regulating gut microbiota composition (Cao et al., 2025). Additionally, kudzu starch had a lower estimated glycemic index value than other starches (Ge et al., 2014).

### 3.5 Worldwide distribution

**FIGURE 2:**
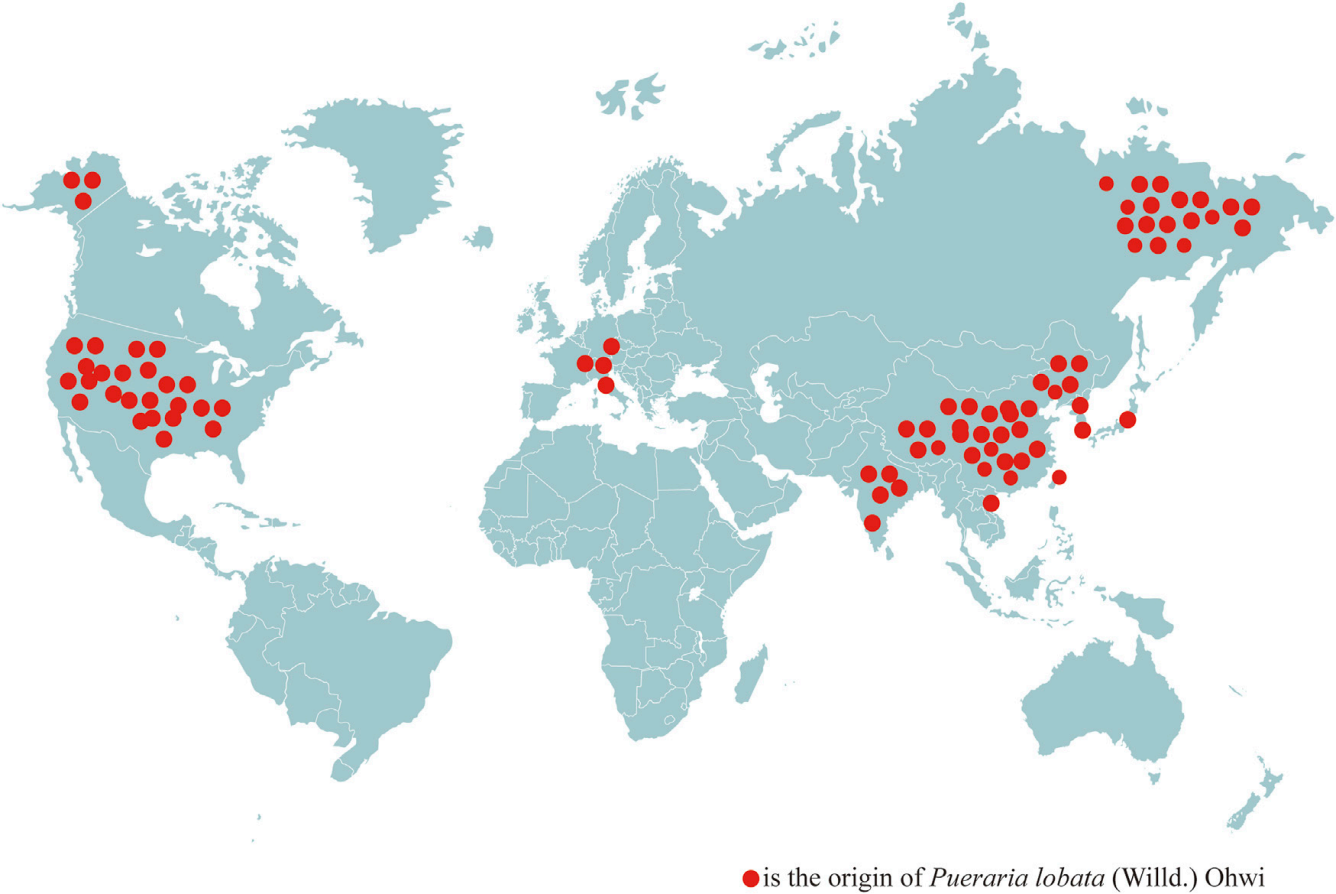
Worldwide distribution of *Pueraria lobata* (Willd.) Ohwi.

Countries other than China have recorded the use of PL in their herbal works. For example, *Da He Ben Cao*, written in Japan at the beginning of the Edo period (approximately 1603 A.D.), clearly records that the vine of PL can be used as a rope, the skin of PL can be used as a cloth, mourning clothes, *etc.*, the leaves of PL can be used as fodder for horses, the powder of PL can quench thirst, stop diarrhea, and treat thirst, and the flowers of PL can quench alcohol. This indicates that the edible and medicinal functions of PL have been used for hundreds of years in many Asian countries. Combined with the *Materia Medica* examination, it can be seen that the medicinal and edible properties of PL have been uninterrupted since ancient times, and throughout the world, PL is mainly distributed in Asia and some parts of Europe and America (Figure 2). PL is found in 13 countries, including China, North Korea (Mun and Mun, 2015), Japan, South Korea, India (Jin et al., 2012), the Russian Far East (Kozhevnikov et al., 2017), Thailand, Vietnam (Nguyen et al., 2009), the United States (Pappert et al., 2000), Switzerland, Austria, Slovenia, and Italy (Follak, 2011). It is widely used in the food and pharmaceutical industries in Asia, whereas PL from Europe and the United States is often treated as an invasive species due to climatic and geographic differences, which has led to its lack of widespread use.

## 4 Phytochemistry

### 4.1 Chemical composition

**FIGURE 3:**
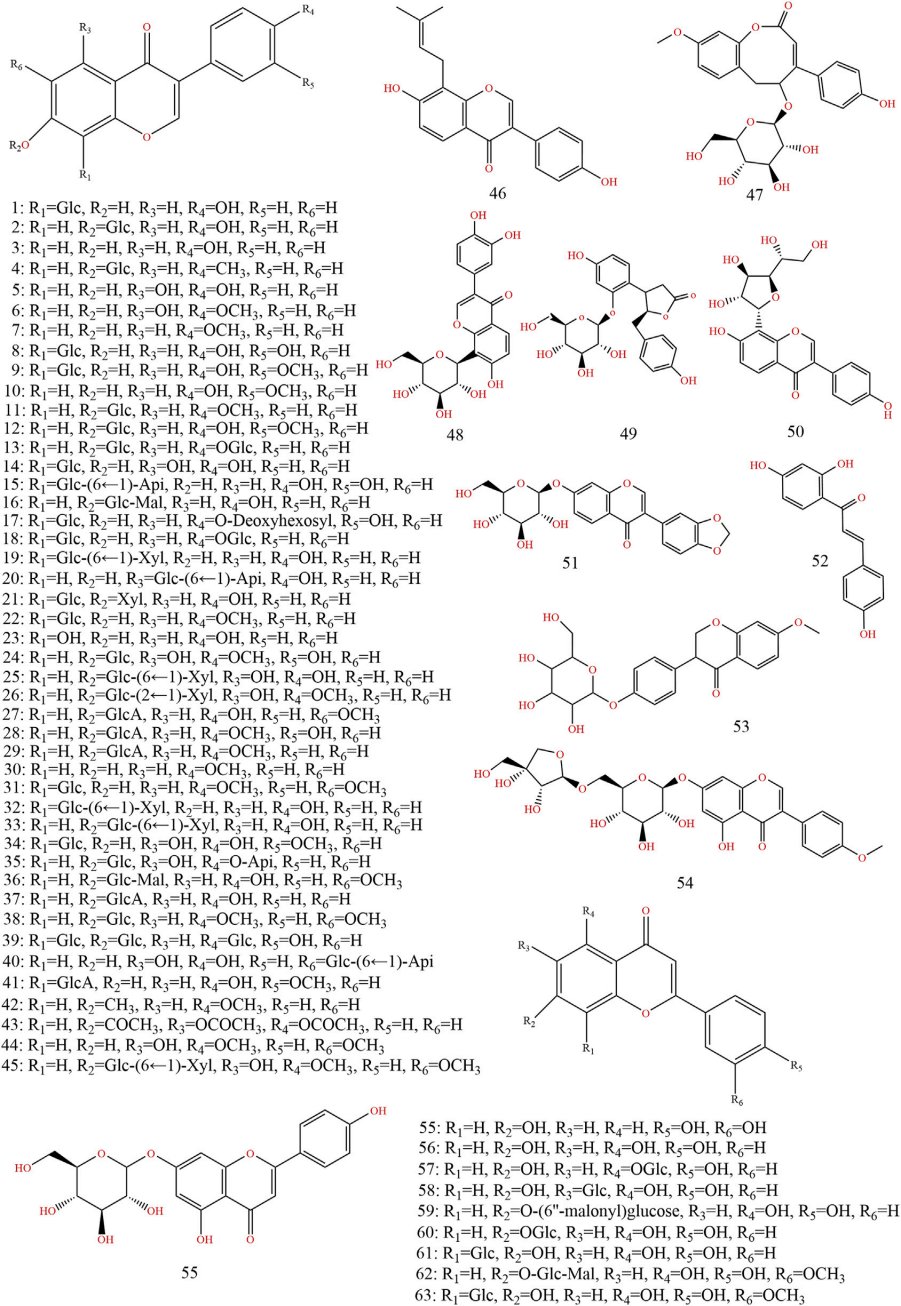
Chemical structures of compounds 1–63 from *Pueraria lobata* (Willd.) Ohwi.

**FIGURE 4:**
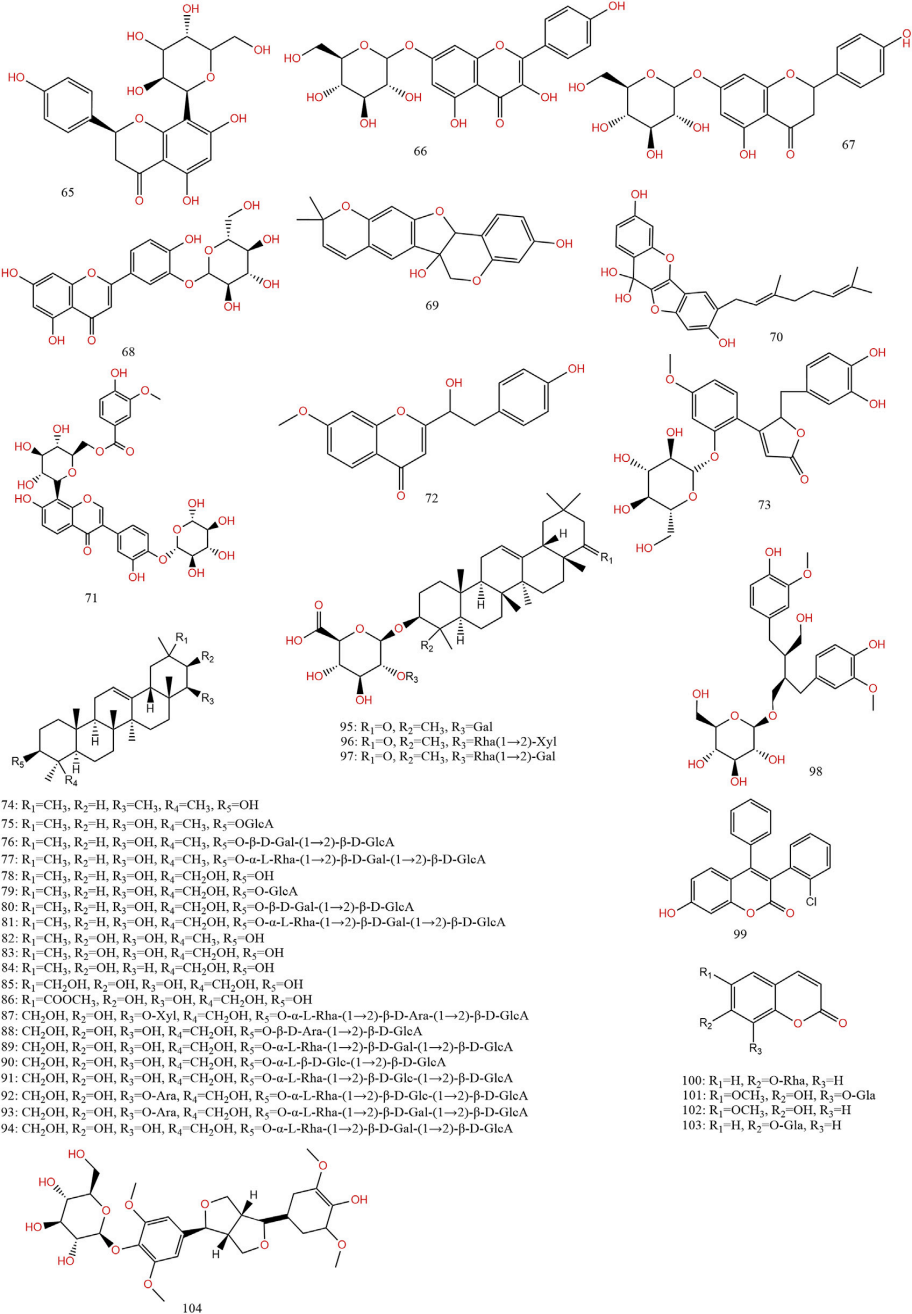
Chemical structures of compounds 64–104 from *Pueraria lobata* (Willd.) Ohwi.

**FIGURE 5:**
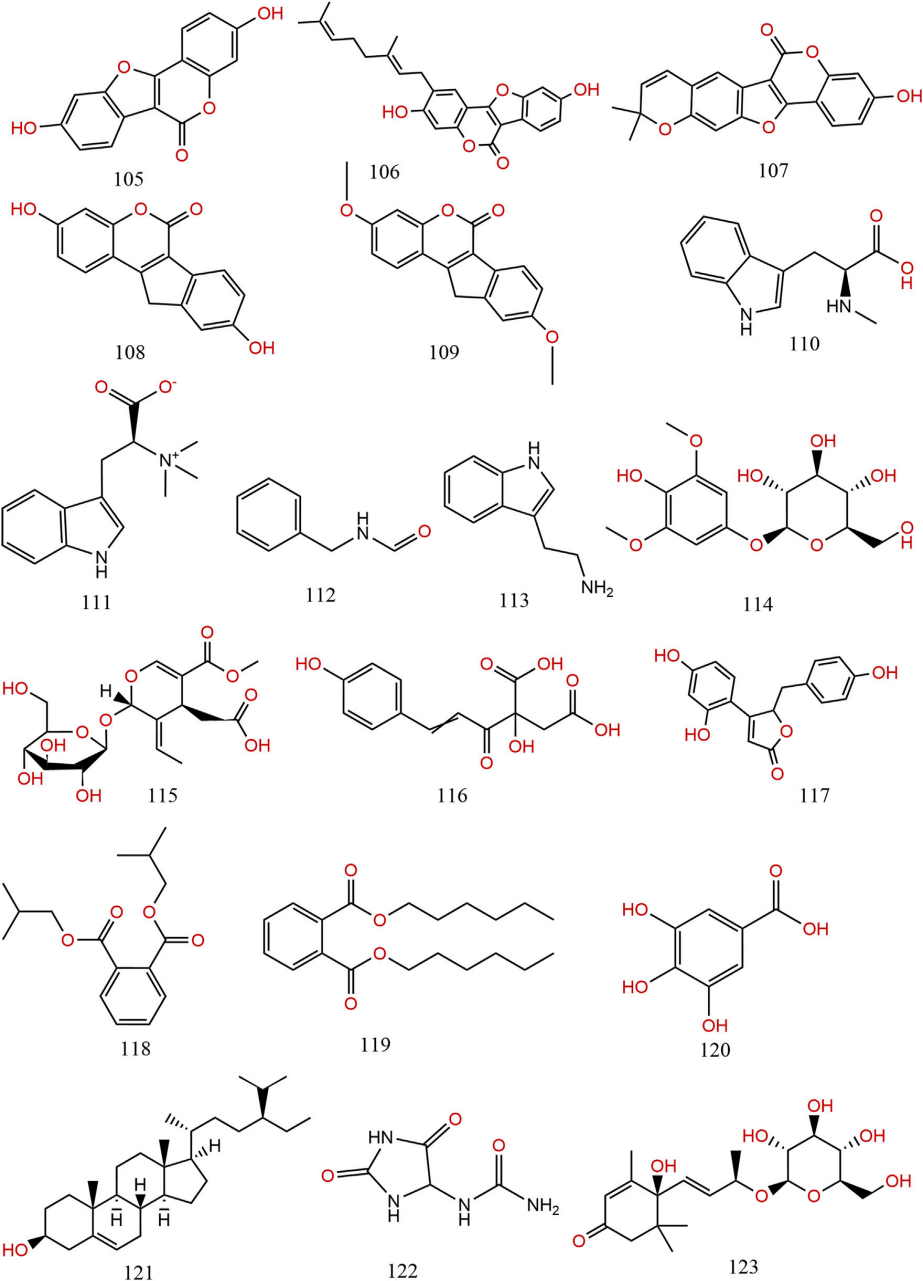
The chemical structures of compounds 105–123 from *Pueraria lobata* (Willd.) Ohwi.

Previous information on the chemical composition of PL is shown in Figures 3–5, which summarize a total of 123 compounds in PL, of which 1–73 are flavonoids, 74–97 are triterpenoids, 98–109 are lignans and coumarins, 110–113 are alkaloids, and 114–123 are others (Table 4).

**TABLE 4:** Chemical composition of *Pueraria lobata* (Willd.) Ohwi.

No.	Compound	Chemical formula	Reference
1	Puerarin	C_21_H_20_O_9_	Kinjo et al. (1987)
2	Daidzin	C_21_H_20_O_9_	Kinjo et al. (1987)
3	Daidzein	C_15_H_10_O_4_	Kinjo et al. (1987)
4	Genistin	C_21_H_20_O_10_	Kinjo et al. (1987)
5	Genistein	C_15_H_10_O_5_	Kinjo et al. (1987)
6	Biochanin A	C_16_H_12_O_5_	Rong et al. (1998)
7	Formononetin	C_16_H_12_O_4_	Kinjo et al. (1987)
8	3′-Hydroxypuerarin	C_21_H_20_O_10_	Chi J et al. (2007)
9	3′-Methoxypuerarin	C_22_H_22_O_10_	Chi J et al. (2007)
10	3′-Methoxydaidzein	C_16_H_12_O_5_	Li et al. (2010)
11	Ononin	C_22_H_22_O_9_	Liu et al. (2012)
12	3′-Methoxydaidzin	C_22_H_22_O_10_	Liu et al. (2012)
13	Daidzein7,4′-O-diglucoside	C_27_H_30_O_14_	Wang et al. (2016)
14	Genistein-8-C-glucoside	C_21_H_20_O_10_	Fang et al. (2006)
15	Daidzein-8-C-apiosyl(1→6)glucoside	C_26_H_30_O_14_	Jin et al. (2012)
16	Malonyldaidzin	C_24_H_22_O_12_	Reppert et al. (2008)
17	3′-Hydroxypuerarin-4′-O-deoxyhexoside	C_27_H_30_O_16_	Rong et al. (1998)
18	Puerarin-4′-O-glucoside	C_27_H_30_O_14_	Nguyen et al. (2009)
19	Puerarin 6″-O-xyloside	C_26_H_28_O_13_	Li et al. (2016a)
20	Daidzein-8-C-apiosyl (1→6)-glucoside	C_26_H_28_O_13_	Jin et al. (2012)
21	Puerarin 7-O-xyloside	C_26_H_28_O_13_	Jiang et al. (2008)
22	4′-Methoxypuerarin	C_22_H_22_O_9_	Sun et al. (2008)
23	8-Hydroxydaidzein	C_15_H_10_O_5_	Piegholdt et al. (2014)
24	Pratensein-7-O-β-D-glucopyranoside	C_22_H_22_O_11_	Wang et al. (2024a)
25	6″-β-D-xylose genistin	C_26_H_28_O_14_	Wang et al. (2024a)
26	Glycyroside	C_27_H_30_O_13_	Zhang et al. (2023b)
27	6″-O-Acetylglycitin	C_23_H_22_O_10_	Tanaka et al. (2011)
28	Calycosin-7-O-β-D-(6″- acetyl)-glucoside	C_24_H_24_O_11_	Wang et al. (2024a)
29	6″-O-Acetylononin	C_24_H_24_O_10_	Wang et al. (2024a)
30	Calycosin	C_16_H_12_O_5_	Wagle et al. (2019)
31	Volubilinin	C_22_H_22_O_10_	Zhang et al. (2023b)
32	Mirificin	C_26_H_28_O_13_	Sun et al. (2019b)
33	Ambonin	C_26_H_28_O_13_	Zhang et al. (2023b)
34	Dalpanitin	C_22_H_22_O_11_	Zhang et al. (2023b)
35	Genistein 7-O-glucoside-4′-O-apioside	C_26_H_28_O_14_	Yan et al. (2013)
36	6″-O-malonylglycitin	C_25_H_24_O_13_	Tanaka et al. (2011)
37	6″-O-Acetyl daidzein	C_23_H_22_O_10_	Delmonte et al. (2006)
38	Wistin	C_23_H_24_O_10_	Hsiou-Yu et al. (2004)
39	3′-Hydroxypuerarin-7,4′-O-glucoside	C_33_H_40_O_19_	Jiang et al. (2005)
40	Genistein 6-C-α-d-apiofuranosyl-(1 → 6)-β--glucopyranoside	C_26_H_28_O_15_	Li et al. (2011)
41	3′-Methoxy-6″-O-acetylpuerarin	C_21_H_20_O_12_	Congbing et al. (2006)
42	4′,7-Dimethoxyisoflavone	C_17_H_14_O_4_	kayano et al. (2012)
43	5-(Acetyloxy)-3-4-(acetyloxy)phenyl-4-oxo-4H-chromen-7-yl acetate	C_21_H_16_O_8_	Tungmunnithum (2020)
44	Irisolidone	C_17_H_14_O_6_	Tungmunnithum (2020)
45	Kakkalide	C_28_H_32_O_15_	Tungmunnithum (2020)
46	8-Prenyldaidzein	C_20_H_18_O_4_	Sankawa et al. (1995)
47	Pueroside C	C_24_H_26_O_10_	Zhang et al. (2019b)
48	3′-Hydroxyneopuerarin A	C_21_H_20_O_10_	Hu et al. (2020)
49	Kuzubutenolide A	C_23_H_24_O_10_	Hirakura et al. (1997)
50	Neopuerarin A	C_21_H_20_O_9_	Zhang (2010)
51	Rothindin	C_22_H_20_O_10_	Wang et al. (2024b)
52	Isoliquiritigenin	C_15_H_12_O_4_	Kinjo et al. (1987)
53	Isoononin	C_22_H_22_O_9_	Ahn et al. (2019)
54	Lanceolarin	C_27_H_30_O_14_	Wang and Yang (2017)
55	3′,4′,7-Trihydroxyflavone	C_15_H_10_O_5_	Shang et al. (2021)
56	Apigenin	C_15_H_10_O_5_	Keung (1993)
57	Apigenin-5-O-glucoside	C_21_H_20_O_10_	Shang et al. (2021)
58	Apigenin-6-C-glucoside	C_21_H_20_O_10_	Rong et al. (1998)
59	Apigenin-7-O-(6″-malonyl)glucoside	C_24_H_22_O_13_	Shang et al. (2021)
60	Apigenin-7-O-glucoside	C_21_H_20_O_10_	Park et al. (1995)
61	Apigenin-8-C-glucoside	C_21_H_20_O_10_	Rong et al. (1998)
62	Chrysoeriol-7-O-(6″-malonyl)glucoside	C_25_H_24_O_14_	Shang et al. (2021)
63	Chrysoeriol-8-C-glucoside	C_22_H_12_O_11_	Shang et al. (2021)
64	Apigenin-7-O-(6″-acetyl)glucoside	C_23_H_22_O_11_	Shang et al. (2021)
65	Isohemiphloin	C_21_H_22_O_10_	Shang et al. (2021)
66	Kaempferol-7-O-glucoside	C_21_H_19_O_11_	Fu et al. (2023)
67	Naringenin-7-O-glucoside	C_21_H_22_O_10_	Wang et al. (2016)
68	Luteolin-3′-O-glucoside	C_21_H_20_O_11_	Braune and Blaut (2011)
69	Tuberosin	C_20_H_18_O_5_	Kim et al. (2006)
70	Lobatflavate	C_30_H_18_O_10_	Wang et al. (2017b)
71	(3-Methoxy-4-hydroxybenzoyl)-3′ hydroxypuerarin-4′-O-glucoside	C3_4_H_34_O_18_	Wang et al. (2024a)
72	Lobatchrosin	C_17_H_14_O_5_	Wang et al. (2017b)
73	3′-Hydroxy pueroside C	C_24_H_26_O_11_	Sook Kim et al. (2014)
74	Sophoradiol	C_30_H_50_O_2_	Kinjo et al. (1988)
75	Sophoradiol monoglucuronide	C_36_H_62_O_9_	Oh et al. (2000)
76	Kaikasaponin Ⅰ	C_42_H_68_O_13_	Oh et al. (2000)
77	Kaikasaponin Ⅲ	C_48_H_78_O_17_	Oh et al. (2000)
78	Soyasapogenol B	C_30_H_50_O_3_	Oh et al. (2000)
79	Soyasapogenol B monoglucuronide	C_36_H_58_O_9_	Oh et al. (2000)
80	Soyasaponin Ⅲ	C_42_H_68_O_14_	Oh et al. (2000)
81	Soyasaponin Ⅰ	C_48_H_78_O_18_	Oh et al. (2000)
82	Cantoniensisitriol	C_30_H_50_O_3_	Kinjo et al. (1985)
83	Soyasapogenol A	C_30_H_50_O_4_	Arao et al. (1995)
84	Kudzusapogenol C	C_30_H_50_O_3_	Arao et al. (1995)
85	Kudzusapogenol A	C_30_H_50_O_5_	Wong et al. (2011)
86	Kudzusapogenol B methylester	C_31_H_50_O_6_	Kinjo et al. (1985)
87	Kudzusaponin A1	C_52_H_84_O_23_	Arao et al. (1997)
88	Kudzusaponin A2	C_42_H_68_O_16_	Arao et al. (1997)
89	Kudzusaponin A3	C_48_H_78_O_20_	Arao et al. (1997)
90	Kudzusaponin A4	C_42_H_68_O_16_	Arao et al. (1997)
91	Kudzusaponin A5	C_48_H_78_O_20_	Arao et al. (1997)
92	Kudzusaponin SA4	C_48_H_74_O_20_	Arao et al. (1997)
93	Kudzusaponin SB1	C_54_H_92_O_26_	Arao et al. (1997)
94	Soyasaponin A3	C_48_H_78_O_19_	Arao et al. (1997)
95	Kakkasaponin II	C_42_H_66_O_13_	Lu et al. (2013b)
96	Kakkasaponin III	C_48_H_78_O_17_	Lu et al. (2013b)
97	Phaseoside IV	C_48_H_76_O_17_	Lu et al. (2013a)
98	(−)-Secoisolariciresinol 4-O-glucoside	C_26_H_36_O_11_	Shang et al. (2021)
99	3-(2′-Chlorophenyl)-7-hydroxy-4-phenylcoumarin	C_21_H_13_ClO_3_	Shang et al. (2021)
100	7-Hydroxycoumarin-O-rhamnoside	C_15_H_18_O_8_	Shang et al. (2021)
101	Fraxetin-8-O-glucoside	C_16_H_18_O_10_	Shang et al. (2021)
102	Scopoletin	C_10_H_8_O_4_	Shang et al. (2021)
103	Skimmin	C_15_H_16_O_8_	Shang et al. (2021)
104	Syringaresinol-4′-O-glucoside	C_28_H_40_O_13_	Cheng et al. (2022)
105	Coumestrol	C_15_H_8_O_5_	Kinjo et al. (1987)
106	Puerarol	C_25_H_24_O_5_	Rong et al. (1998)
107	Sophoracoumestan A	C_20_H_14_O_5_	Li et al. (2010)
108	Puerarol dimethylether	C_16_H_10_O_4_	Ohshima et al. (1988)
109	Psorali dindimetherylether	C_18_H_14_O_4_	Ohshima et al. (1988)
110	Abrine	C_12_H_14_N_2_O_2_	Ramawat and Mèrillon (2023)
111	Hypaphorine	C_14_H_18_N_2_O_2_	Shang et al. (2021)
112	N-Benzylformamide	C_8_H_9_NO	Shang et al. (2021)
113	Tryptamine	C_10_H_12_N_2_	Shang et al. (2021)
114	Koaburaside	C_14_H_20_O_9_	Shang et al. (2021)
115	Oleoside 11-methyl ester	C_17_H_24_O_11_	Shang et al. (2021)
116	p-Coumaroylmalic acid	C_13_H_12_O_7_	Shang et al. (2021)
117	Puerol A	C_17_H_14_O_5_	Wu et al. (2013)
118	Diisobutyl phthalate	C_16_H_22_O_4_	Dong et al. (2024)
119	Dihexyl phthalate	C_20_H_30_O_4_	Li et al. (2010)
120	Gallic acid	C_7_H_6_O_5_	Wei et al. (2020)
121	β-Sitosterol	C_29_H_50_O	Wei et al. (2020)
122	Allantoin	C_4_H_6_N_4_O_3_	Lynd and Ansman (1990)
123	Roseoside	C_19_H_30_O_8_	Lim (2016)

### 4.2 Biosynthetic and metabolic pathways

#### 4.2.1 Biosynthetic pathways

**FIGURE 6:**
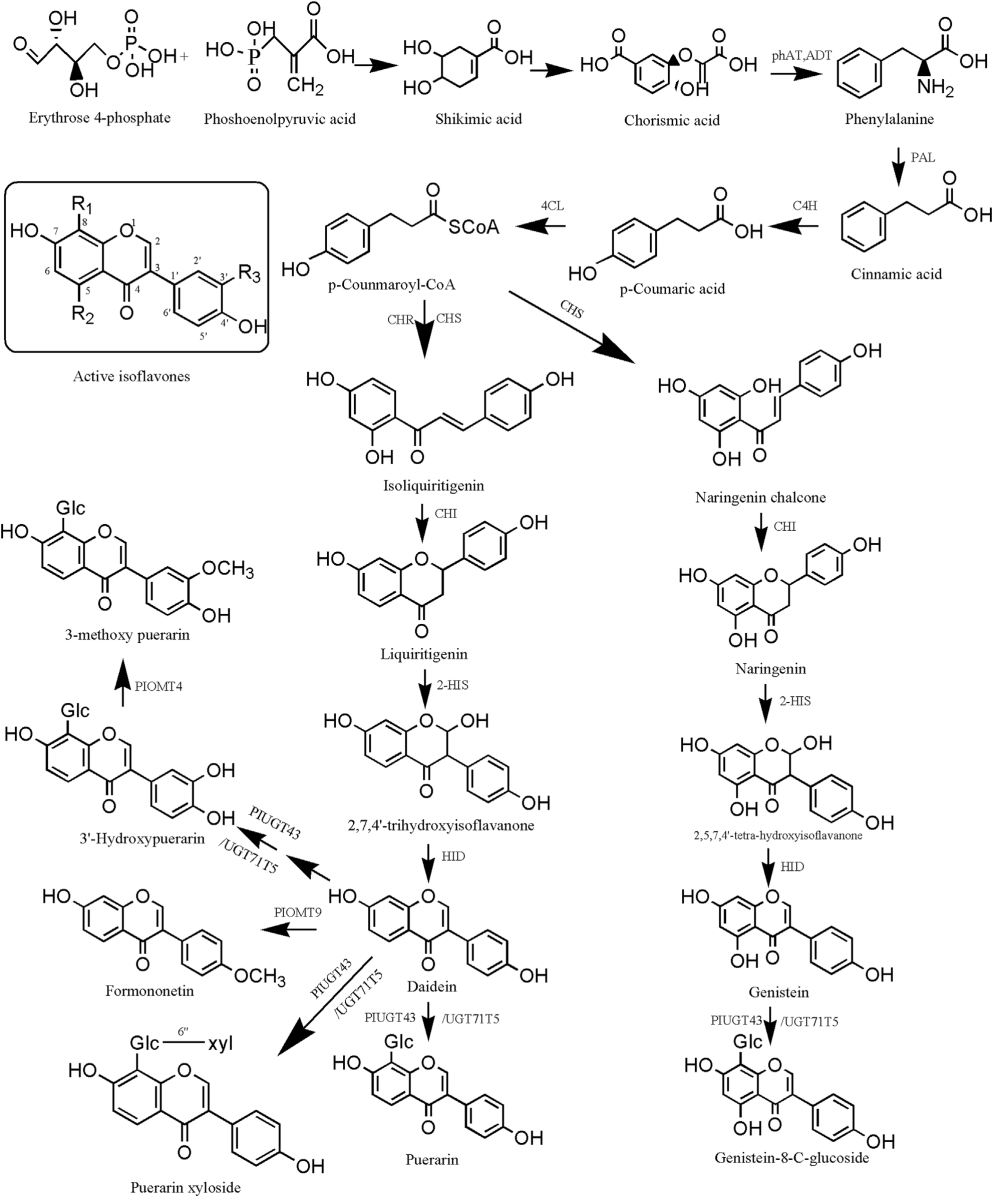
Biosynthesis pathway of isoflavonoid metabolites in *Pueraria lobata* (Willd.) Ohwi. phAT, plant phosphotransacetylase; ADT, plant aronic acid dehydratase; PAL, phenylalanine ammonia-lyase; C4H, cinnamate 4-hydroxylase; 4CL, 4-coumarate-CoA ligase; CHR, chalcone reductase; CHS, chalcone synthase; CHI, chalcone isomerase; HID, 2-hydroxyisoflavanone dehydratase.

The biosynthetic pathways of isoflavonoid metabolites in PL mainly include the mangiferolic acid, phenylpropionic acid, and puerarin pathways. After the production of p-coumaroyl-CoA, isoliquiritigenin was catalyzed by CHS and CHR, liquiritigenin was produced under the catalysis of CHI, 2,7,4′-trihydroxyisoflavanone was produced under the catalysis of 2-HIS, and daidzein wasp roduced under the catalysis of HID. Daidzein can produce puerarin and puerarin xyloside via PIUGT43. 3′-Hydroxydaidzein produces 3′-hydroxypuerarin via PIUGT43, and daidzein produces formononetin via PIOMT9. 3′-Hydroxypuerarin can be converted to 3-methoxy puerarin when catalyzed by PIOMT4. In addition, p-coumaroyl-CoA catalyzed by CHS directly produced naringenin chalcone, naringenin chalcone catalyzed by CHI produced naringenin, naringenin catalyzed by 2-HIS produced 2,5,7,4′-tetra- hydroxyisoflavanone, 2,5,7,4′-tetra-hydroxyisoflavanone catalyzed by HID produced genistein, and post-genistein can undergo the action of PIUGT43 or UGT71T5 to produce genistein-8-C-glucoside (Figure 6).

From the above, it can be seen that the active isoflavone metabolites are the common skeleton with the presence of a wide range of isoflavonoid metabolites in PL, daidzein, puerarin, genistein, and so on. They are the mass signature metabolites. 6-C, 8-C, 3′-C, and 4′-C are the active sites of methylation and glycosylation that are susceptible to them (Li and Zhang, 2023). Of these, there are two theories for glycosylation at the 8-C position. One theory is that it is regulated through the PIUGT43 gene (Liga and Paul, 2024). The second theory is regulation through the UGT71T5 gene (Adolfo et al., 2022). Li et al. (2021) investigated the response of PL to selenium (Se) stimulation and found that five miRNAs targeting nine regulatory protein genes, which may be involved in the biosynthesis of isoflavonoids. Li et al. (2016b) found that PlOMT9 plays an important role in the biosynthesis of formononetin. PlOMT4 is a key enzyme in the 3′O-methylation of active isoflavones to enhance the lipophilicity of similar metabolites (Li et al., 2016c).

#### 4.2.2 Metabolic pathways

**FIGURE 7:**
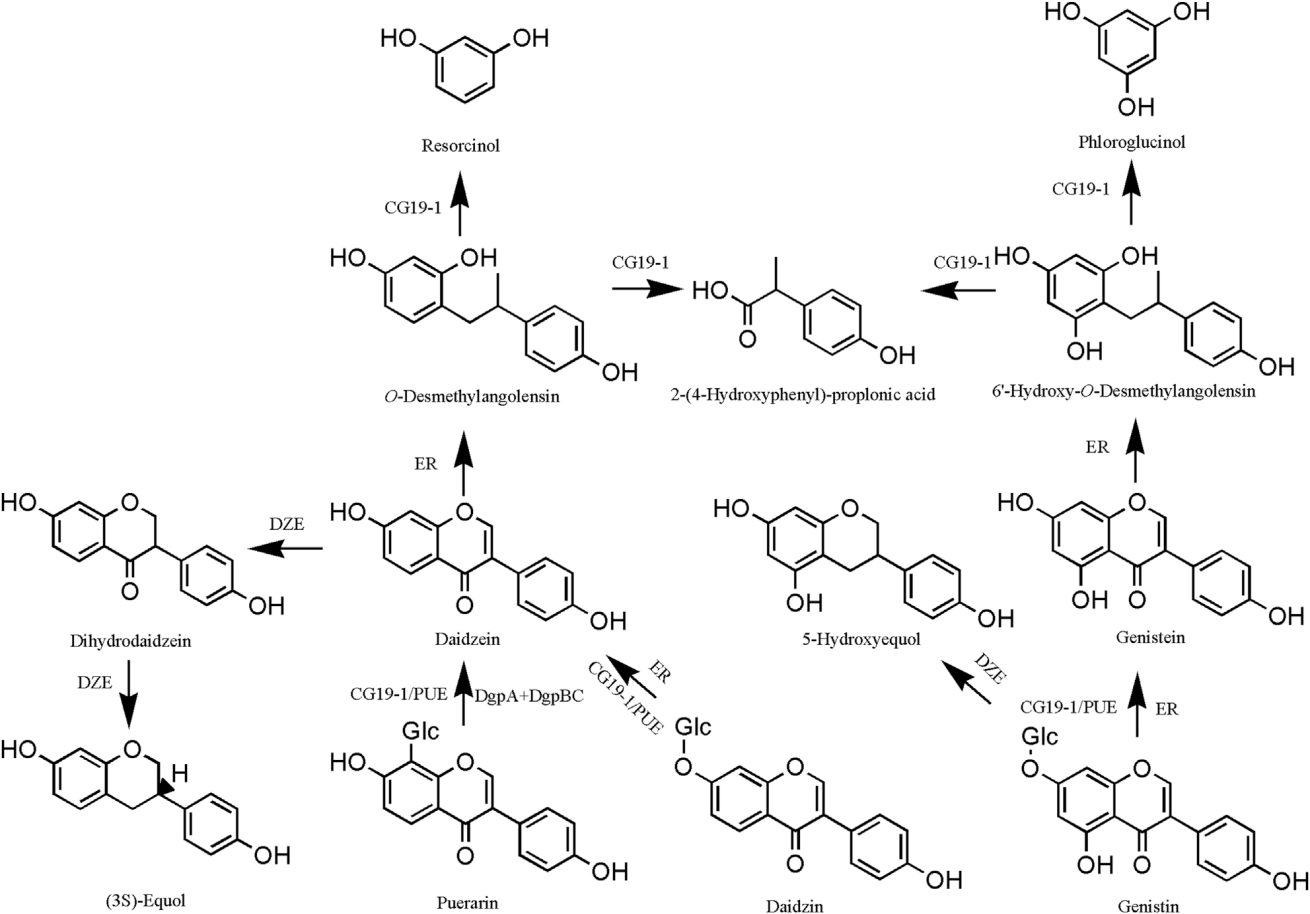
Intestinal metabolism of *Pueraria lobata* (Willd.) Ohwi isoflavones. DgpA, GenBank, BBG22493.1; DgpBC, PDBID, EM Map EMD-30808; PUE, 1.96 0.09 optical density (O.D.); DZE, 0.30 0.02 O.D.

Isoflavones are susceptible to oxidation in the presence of intestinal endophytes to produce ethylphenol derivatives. The isoflavones in PL undergo two main types of reactions in the gut. In the case of puerarin, for example, one is the generation of daidzein by strain CG19-1/PUE or DgpA + DgpBC bacteria (Choi et al., 2023). Subsequently, daidzein was converted to O-desmethylangolensin by *Eubacterium ramulus* (ER), and O-desmethylangolensin was catalyzed by CG19-1 to produce resorcinol, 2-(4-hydroxyphenyl)-propionic acid (Braune and Blaut, 2011). The second is the generation of dihydrodaidzein from daidzein catalyzed by key bacteria such as PUE and DZE, which ultimately generates (3S)-equol (Jin et al., 2008) (Figure 7). Puerarin effectively attenuated M1-like macrophage activation mediated through *Akkermansia muciniphila*-derived protein Amuc_2172, consequently ameliorating pathological alterations in the dextran sulfate sodium (DSS) model (Tao et al., 2024b). The metabolites demonstrate preventive effects against high-fat-diet-induced obesity by inhibiting *A. muciniphila* activity (Wang et al., 2019b; Yang C. W. et al., 2024).

**FIGURE 8:**
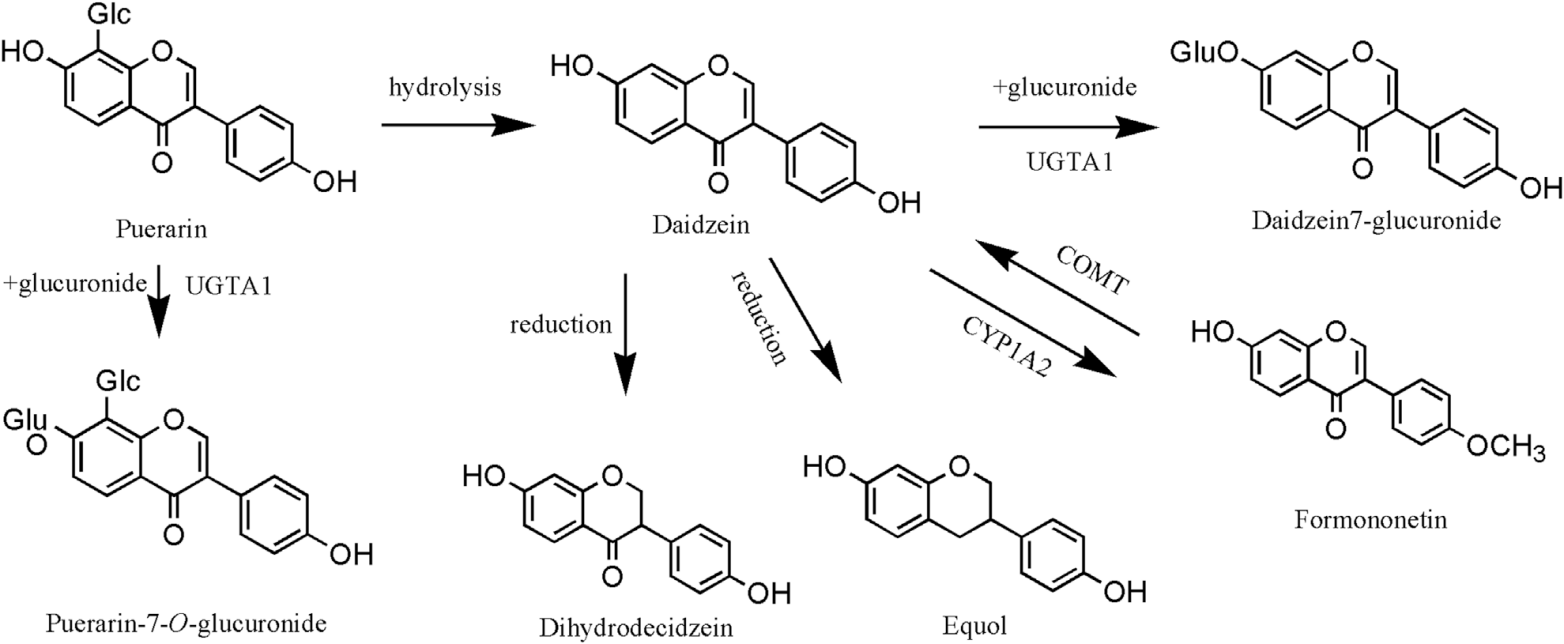
Liver metabolism of *Pueraria lobata* (Willd.) Ohwi isoflavones. UGTA1, UDP-glucuronosyltransferase 1A1; COMT, catechol-O-methyltransferase, Gene ID 1312; CYP1A2, cytochrome P450 family 1 subfamily A polypeptide 2.

In the liver, the isoflavones of PL often undergo glucuronidation, methylation, and reduction reactions. The hepatic metabolism of isoflavones was elucidated using daidzein and puerarin as examples (Figure 8). First, puerarin undergoes glucuronidation catalyzed by UGTA1 (UDP-glucuronosyltransferase 1A1: Gene ID 54658) to produce puerarin-7-O-glucuronide, or it undergoes hydrolysis to produce daidzein (Luo et al., 2012). In addition, daidzein is converted to formononetin catalyzed by CYP1A2 (cytochrome P450 family 1 subfamily A polypeptide 2: Gene ID 1543) enzyme and may also produce dihydrodaidzein, equol, and others (Atherton et al., 2006). It has been confirmed that the products after glucuronidation and reduction reactions are one of the important material bases for the action of puerarin, so the research on the hepatic metabolism of the isoflavones of PL is of great significance. Furthermore, puerarin demonstrates targeted therapeutic effects on hepatocytes. As evidenced by Fang et al. (2024), this metabolite facilitates macrophage phenotype switching from M1 to M2 type through ULK-1 upregulation and PAI-1 downregulation. This mechanism specifically restores hepatocyte autophagy homeostasis and ameliorates pathological alterations.

## 5 Pharmacology

**FIGURE 9:**
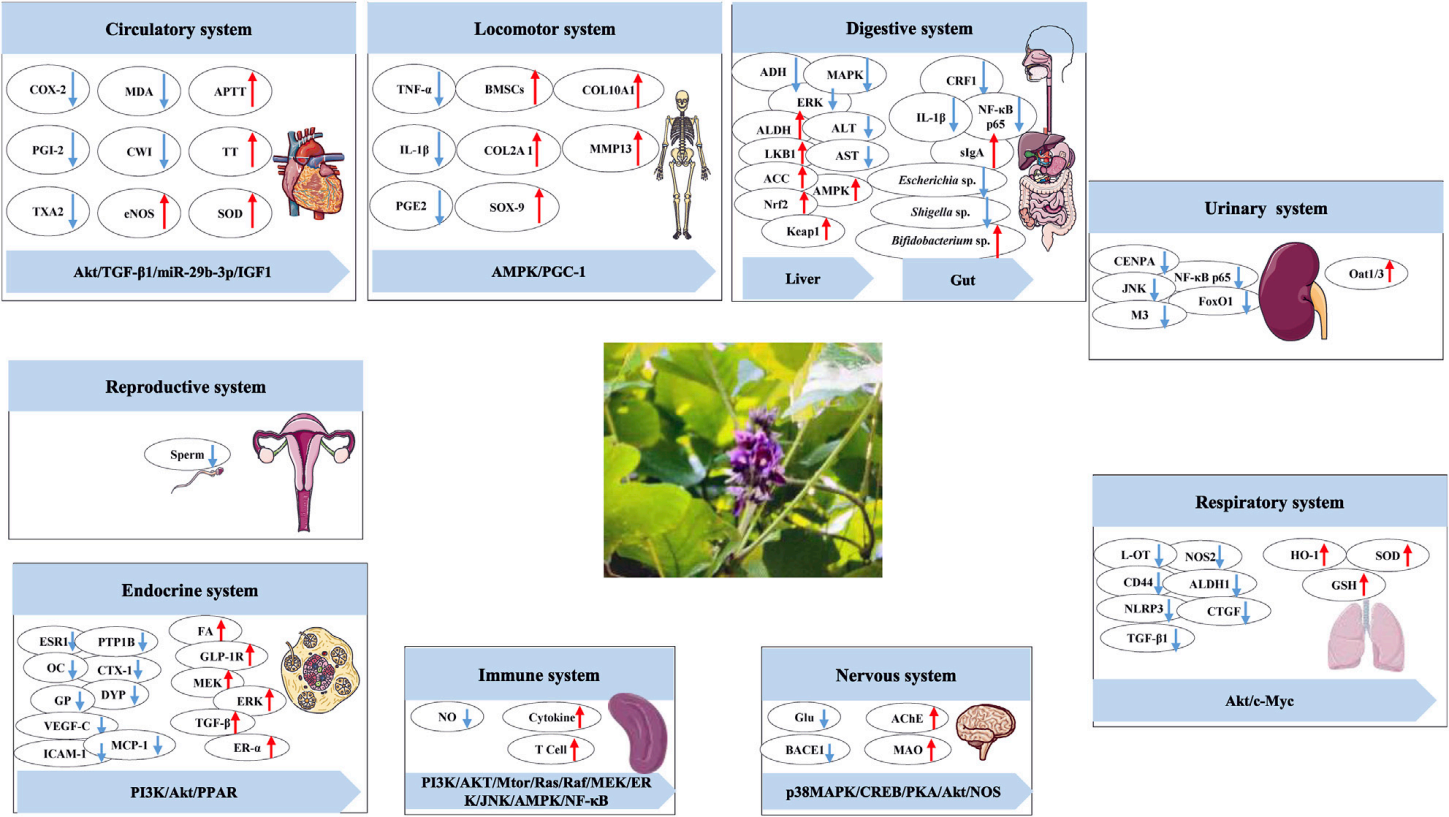
Biological activities of *Pueraria lobata* (Willd.) Ohwi.

PL has a wide range of biological activities, which are summarized according to the classification of the nine major systems of the human body in biology. The results are shown in Figure 9 and Table 5–8.

**TABLE 5:** Bioactivities of extracts or compounds from *Pueraria lobata* (Willd.) Ohwi in the circulatory and locomotor systems.

Pharmacological effect	Cell line (s)/models	Type of extract/compounds	Dose range	Minimal active concentration	Duration	Control	Effect on cellular target	Reference
Antithrombotic	SD rats	Aqueous extract	1.575–6.3 g/kg	Not stated	2 weeks	Negative and positive (aspirin)	COX-2↓, PGI2↓, TXA2↓, MDA↓, eNOS↑, SOD↑, APTT↑, TT↑	Wang et al. (2023b)
Anti-myocardial hypoxic injury	NRC cells	Puerarin (1)	50–200 μmol/L	Not stated	8 h	Negative	LC-3Ⅱ/LC-3Ⅰ↓, p62↑, pAkt/Ak↑	Tang et al. (2017)
Anti-myocardial fibrosis	KM mice	Puerarin (1)	600–1,200 mg/kg	Not stated	40 days	Negative	CWI↓, TGF-β1↓, NF-κB-p65↓, PPAR α/Γ↑	Chen et al. (2012)
Anti-atherosclerosis	HVSMC cells and apolipoprotein E knockout mice (ApoE^−/−^ mice)	Puerarin (1)	50–200 μmol/kg/d	Not stated	4 weeks	Negative and positive (fluvastatin)	miR-29b-3p, IGF1↑	Li et al. (2022)
Anti-infarction	Wistar rats	Methanol extract	0–400 mg/kg	Not stated	15 days	Negative	PI3↑, Akt↑	Li and Vijayalakshmi (2023)
Anti-infarction	SD rats	Ethanol extract	0–424 mg/kg/d	Not stated	2 weeks	Negative and positive (isosorbide mononitrate SR tablets)	CYP7A1↑	Zhang et al. (2023a)
Anti-osteoarthritis	SD rats	Puerarin (1)	100–200 mg/kg	100 mg/kg	14 days	Negative	AMPK, PGC-1↑	Wang et al. (2018b)
Antibacterial bone disease	RAW264.7 cells and ICR male mice	Puerarin (1)	*In vitro*: 10–50 μmol/L; *in vivo*: 0–1 mg/kg/d	Not stated	48 h	Negative	TNF-α↓, IL-1β↓, PGE 2↓, TRAP↓, MMP-9↓	Zhang et al. (2016)
Anti-osteoarthritis	BMSCs	Puerarin (1)	0.01–1 μmol/L	Not stated	3–7 days	Negative	COL2A 1↑, COL10A 1↑, MMP13↑, SOX-9↑	Dong et al. (2022)

**TABLE 6:** Bioactivities of extracts or compounds from *Pueraria lobata* (Willd.) Ohwi in the digestive system.

Pharmacological effect	Cell line (s)/models	Type of extract/compounds	Dose range	Minimal active concentration	Duration	Control	Effect on cellular target	Reference
Anti-non-alcoholic fatty liver disease	HepG2 cells, Wistar rats	70% ethanol extract	*In vitro*: 50–200 μg/mL; *in vivo*: 50–200 mg/kg	Not stated	*In vitro*: 24–48 h; *in vivo*: 4 weeks	Negative and positive (*in vitro*: vitamin C; *in vivo*: simvastatin)	Keap1↑, Nrf2↑, HO-1↑	Dong et al. (2024)
Antioxidant and repair effects	HepG2 Cells	Protein	0.1–100 μg/mL	50 μg/mL	24 h	Negative	Keap1↑, Nrf2↑, GSH↑, CAT↑, SOD↑, ROS↓, MDA↓	Pang et al. (2023)
Anti-hepatization	C57BL/6J mice	Puerarin (1) and genistein (5)	0–0.3 mmol/kg	Not stated	8 weeks	Negative	MAPK↓, ERK↓	Li et al. (2024)
Anti-alcoholism	C57BL/6J mice	Exosome-like nanovesicles	2–50 mg/kg	10 mg/kg	6–10 h	Negative	ALDH↑, ADH↓	Zhang et al. (2024b)
Anti-non-alcoholic fatty liver disease	C57BL/6J mice	Starch	0–400 mg/kg	Not stated	8 weeks	Negative and positive (silymarin)	IL-6↓, TNF-α↓, ALT↓, AST↓, TC↓, TG↓	Yang et al. (2022)
Anti-alcoholic liver disease	KM mice	Polysaccharide	100–400 mg/kg	Not stated	21 days	Negative (normal saline)	ALT↓, AST↓, TC↓, TG↓	Cao et al. (2024)
Antibiotic-associated diarrhea	Mice	Polysaccharide	0–12.5 mg/kg	Not stated	14 days	Negative	Isovaleric acid↓, *Oscillospira*↑, *Anaerotruncus*↑	Chen et al. (2020)
Anti-colitis	C57BL/6J mice	Starch	0–200 mg/kg	Not stated	29 days	Negative	NF-κB p65↓, IL-1β↓	Yang et al. (2023)
Anti-intestinal mucosal damage	ICR mice	Polysaccharide	0.4–1.2 g/kg	Not stated	7 days	Negative	sIgA↑, CD4+↑, CD8+ ↑, ZO-1↑, Occludin↑, SOD↑, MDA↓	Cai et al. (2022b)
Anti-diarrhea	Dairy calves	Polysaccharide	0–400 mg/kg	Not stated	5 days	Negative	IL-1β↓, TNF-α↓, MDA↓, Proteobacteria↓, Fusobacteria↓, SOD↑	Shen et al. (2023)
Anti-ulcerative colitis	BALB/c mice	Puerarin (1)	10–50 mg/kg	Not stated	7 days	Negative	NF-κB↓, Nrf2↑	Jeon et al. (2020)

**TABLE 7:** Bioactivities of extracts or compounds from *Pueraria lobata* (Willd.) Ohwi in the respiratory and urinary systems.

Pharmacological effect	Cell line (s)/models	Type of extract/compounds	Dose range	Minimal active concentration	Duration	Control	Effect on cellular target	Reference
Anti-pulmonary fibrosis	SD rats	Aqueous extract	3.2–6.4 g/kg	Not stated	28 days	Negative	NOS2↓, TNF-α↓, MMP-9↓, L-OT	Du et al. (2024)
Anti-airway inflammation	BALB/c mice	Puerarin (1)	10–20 mg/kg	Not stated	48 h	Negative	IL-4↓, IL-5↓, IL-13↓, Eotaxin-3↓, IFN-γ↑	Wang et al. (2015)
Anti-acute lung injury	RAW264.7 cells and C57BL/6J mice	Puerarin (1)	*In vitro*: 0–20 μmol/;*,* *in vivo*: 0–30 mg/kg	Not stated	*In vitro*: 24 h; *in vivo*: 12 h	Negative	NLRP3↓, HDAC1↓, TNF-α↓, IL-6↓, PP2A↑	Cai et al. (2022a)
Anti-early chronic obstructive pulmonary disease	HSAECS cells and C57BL/6J mice	Puerarin (1)	*In vitro*: 20–40 μmol/L; *in vivo*: 40–80 mg/kg	Not stated	*In vitro*: 1 h; *in vivo*: 6 h	Negative	NOX1, NOX2, NOX4↓, TNF-α↓, IL-6↓, COX-2↓	Zhang et al. (2022)
Anti-pulmonary ischemia–reperfusion injury	New Zealand rabbits	Puerarin (1)	0–30 mg/kg	Not stated	5 h	Negative	Fas↓, FasL↓	Zheng et al. (2015)
Anti-colitis-associated lung inflammation	C57BL/6J mice	Exosome-like nanovesicles	0.5–5 mg/kg	Not stated	7 days	Negative, positive (mesalazine)	IL-6↓, IL-1β↓, TNF-α↓, CCL2↓	Lu et al. (2024)
Anti-compulsive muscle overactivity	Spontaneously hypertensive rats and Wistar Kyoto rats	Aqueous extract	30–300 mg/kg	Not stated	3 weeks	Negative	Improvement of forced urethral muscle overactivity through neurogenic pathways	Zhou et al. (2016)
Anti-renal inflammatory injury	RAW264.7 cells and C57BL/6J mice	Puerarin (1)	*In vitro: 0–50 μmol/*;*,* *in vivo: 50–100 mg/kg*	Not stated	*In vitro*: 24 h; *in vivo*: 3 days	Negative	NF-κB p65↓, JNK↓, FoxO1↓, TLR4↓, MyD88↓	Hu et al. (2024)
Anti-renal injury	Wistar rats	Puerarin (1)	0–50 mg/kg	Not stated	6 days	Negative	Oat 1/3↑	Liu et al. (2018)
Anti-pelvic prolapse	SD rats	Puerarin (1)	0–40 mg/kg	Not stated	15 days	Negative	IL-1	Zhang et al. (2024a)

**TABLE 8:** Bioactivities of extracts or compounds from *Pueraria lobata* (Willd.) Ohwi in the endocrine, nervous, and immune systems.

Pharmacological effects	Cell line (s)/models	Type of extract/compounds	Dose range	Minimal active concentration	Duration	Control	Effect on cellular target	Reference
Anti-diabetic	HepG2 cells and C57BL/6J mice	75% ethanol extract	*In vitro*: 0.0014–0.046 mg/mL; *in vivo*: 250–2000 mg/kg	Not stated	*In vitro*: 24 h; *in vivo*: 2 weeks	Negative	PTP1B↓	Sun et al. (2019a)
Anti-diabetic	C57BL/KsJ-db/db mice	Polysaccharide	0–200 mg/kg	Not stated	5 weeks	Negative	LKB1, AMPK↑, mTOR↓	Wang et al. (2022)
Anti-diabetic	C57BL/6 mice	Puerarin (1)	150–300 mg/kg	Not stated	20 days	Negative	GLP-1R↑	Wang et al. (2020)
Anti-menopausal dysfunction	SD rats	80% ethanol extract	50–200 mg/kg	Not stated	8 weeks	Negative	TG↓, TC↓, ALT↓, AST↓	Oh et al. (2019)
Anti-osteoporosis	SD rats	50% ethanol extract	25–1,600 mg/kg	Not stated	8 weeks	Negative	CTX-1, OC, DYP↓, ER-α↑	Lee et al. (2021)
Anti-osteoporosis	ICR mice	60% ethanol extract	250–500 mg/kg	Not stated	4 weeks	Negative	BMD↑, SULT1E1↑	Cho et al. (2012)
Anti-osteoporosis	MG-63 cells	Puerarin (1)	0.01–1 μmol/L	Not stated	3 days	Negative (DMSO)	OPG, RANKL↓, IL-6↓	Wang et al. (2014)
Anti-osteoporotics	Mice	Puerarin 6″-O-xyloside (19)	20–60 mg/kg	Not stated	12 weeks	Negative	ALP↑, OPG↑, OPG/RANKL↑	Li et al. (2016a)
Anti-osteoporosis	Bone Marrow Mesenchymal Stromal	Puerarin (1)	0–50 μmol/L	Not stated	24 h	Negative (DMSO)	Atg5↓, Atg7↓, BECN1↓	Zhang et al. (2019a)
Anti-osteoporosis	MG-63 and HBL-100 cells	Puerarin (1)	0.01–1 μmol/L	Not stated	3 days	Negative	Bcl-xL↑, MEK↑, ERK↑, PI3K↑, Akt↑	Wang et al. (2013)
Anti-Alzheimer’s disease	SD rats	Puerarin (1)	50–200 mg/kg	Not stated	14 days	Negative	GSH↑, AChE↓, MDA↓	Liu et al. (2021b)
Anti-learning and memory disorders	KM mice	Puerarin (1)	50–150 μg/kg	Not stated	15 days	Negative	AChE↓	Wu et al. (2017)
Anti-hypoxic cerebral edema	SD rats	Puerarin (1)	0–50 mg/mL	Not stated	14 days	Negative	AQP 4↓, NF-κB↓	Wang et al. (2018a)
Boosting immunity	ICR mice	Polysaccharide	*In vitro*: 0.0785–0.5 mg/mL; *in vivo*: 400–1,200 mg/kg	*In vitro*: 0.125 mg/mL; *in vivo*: 400 mg/kg	*In vitro*: 24 h; *in vivo*: 7 days	Negative	IL-2↑, IL-4↑, TNF-α↑, IFN-γ↑	Cai et al. (2023)

### 5.1 Circulatory system

PL has obvious antithrombotic effects, and its aqueous extract was able to reduce cyclooxygenase-2 (COX-2), prostaglandin-I-2 (PGI2), thromboxane A2 (TXA2), and malondialdehyde (MDA). The extract can elevate endothelial nitric oxide synthase (eNOS), superoxide dismutase (SOD), activated partial thromboplastin time (APTT), and thrombin time (TT) in an arteriovenous bypass thrombosis model in rats (Wang S. et al., 2023). Puerarin has a significant inhibitory effect on a hypoxia-induced myocardial ischemia model in mammary rat cardiomyocytes, as evidenced by the reduction of LC-3II/LC-3Ⅰ and the elevation of p62 and protein kinase B (pAkt)/Akt expression (Tang et al., 2017). In addition, puerarin was able to prevent subcutaneous isoprenaline-induced myocardial fibrosis by decreasing cell-wall integrity (CWI), transforming growth factor-beta (TGF-β) 1, nuclear factor kappa-B (NF-κB) -p65, and elevating peroxisome proliferator-activated receptors (PPAR) α/Γ (Chen et al., 2012). Puerarin also inhibits vascular smooth muscle proliferation in atherosclerosis *via* the miR-29b-3p/IGF1 (insulin-like growth factor 1) pathway (Li et al., 2022). This suggests that PL has an irreplaceable effect on the circulatory system, both from a compositional and ancient formulas. A study has confirmed that an extract of PL leaves can improve experimental myocardial infarction in Wistar rats through the (phosphatidylinositol 3-kinase) PI3K/Akt signaling pathway (Li and Vijayalakshmi, 2023). Zhang P. et al. (2023) combined the joint analysis of intestinal flora and bile acid metabolism and found that the ethanol extract of PL was able to effectively improve the bile acid level in rats with acute myocardial infarction by increasing the expression of cholesterol 7 alpha-hydroxylase (CYP7A1) and restoring the diversity of intestinal flora.

### 5.2 Locomotor system

Due to the presence of isoflavonoids such as puerarin, which has been described as a “natural phytoestrogen” (Wang X. et al., 2024), and the fact that estrogens have been shown to promote bone growth and development (Rizzoli and Bonjour, 1997), the motor systemic activity of PL should not be overlooked. Wang L. et al. (2018) found that puerarin could alleviate mitochondrial dysfunction and reduce osteoarthritis in osteoarthritic rats by upregulating the adenosine 5′-monophosphate (AMP)-activated protein kinase (AMPK)/(peroxisome proliferator-activated receptor-γ coactivator) PGC-1 signaling pathway. Zhang et al. (2016) found that puerarin downregulated the mRNA expression of osteoclastogenesis-related genes, anti-tartrate acid phosphatase (TRAP), histone K, and (matrix metalloprotein-9), MMP-9 by inhibiting the production of osteoclast-activating tumor necrosis factor (TNF)-α, interleukin (IL)-1β, and prostaglandin E2 (PGE 2). This informs the prevention and treatment of bacterial bone-destructive diseases. Xin-Ran Dong’s group found that puerarin promotes the proliferation and induces the differentiation of bone marrow mesenchymal stem cells (BMSCs) to chondrocytes by increasing the protein expression of collagen type II alpha 1 chain (COL2A 1), collagen type X alpha 1 chain (COL10A 1), MMP13, and SRY-related high-mobility-group box (SOX)-9, which has potential therapeutic effects in osteoarthritis (Dong et al., 2022).

### 5.3 Digestive system

The main organs of the digestive system involved in the action of PL are the liver and intestines. First, isoflavones and triterpenoid saponins are the important material basis for liver protection of PL (He et al., 2021). Our previous study found that a 70% ethanol extract of PL could improve non-alcoholic fatty liver by changing liver fat accumulation and activating the Kelch-like ECH-associated protein 1 (Keap1)/nuclear respiratory factor 2 (Nrf2)/heme oxygenase (HO)-1 signaling pathway (Dong et al., 2024). In addition, our group also confirmed the functional characteristics of the protein of PL and reduced the accumulation of reactive oxygen species and malondialdehyde after oxidative damage of HepG2 cells by activating the Nrf2/Keap1 signaling pathway, increased superoxide dismutase activity, peroxidase activity, and increased GSH content (Pang et al., 2023). Li et al. (2024) used network pharmacology and experimental validation methods to confirm that puerarin and genistein in PL could increase ferredoxin levels by blocking the MAPK/ERK (extracellular signal-regulated kinase) signaling pathway, thereby attenuating alcohol-induced iron overload, hepatic injury, oxidative stress, and hepatocellular apoptosis. It was found that PL nanovesicles possessed exosomal properties, enhanced acetaldehyde dehydrogenase (ALDH) activity, inhibited alcohol dehydrogenase (ADH) activity, and improved the anti-intoxication and alcohol metabolizing ability of acute alcohol-poisoned mice (Zhang W. et al., 2024). Moreover, it was found that the component promoting alcohol metabolism might not be puerarin, and the key substances and mechanisms need to be explored in depth. The starch of PL can improve non-alcoholic fatty liver disease by adjusting intestinal microecology, providing a basis for its food function (Yang et al., 2022). Water-soluble polysaccharides of PL regulate lipid metabolism by inhibiting oxidative stress and increasing the production of short-chain fatty acids, thereby ameliorating liver injury in mice with acute alcoholic liver disease (Cao et al., 2024).

Chen et al. (2020) found that PL polysaccharides could treat antibiotic-associated diarrhea by attenuating the colonic lesions and dysbiosis of intestinal flora. Furthermore, the starch of PL and its branched chain starches attenuated DSS-induced colitis by decreasing the expression of NF-κB p65 and IL-1β protein levels and correcting gut microbiota disturbances (Yang et al., 2023). Cai G. et al. (2022) found that kudzu polysaccharides significantly improved intestinal mucosal immune function in cyclophosphamide-treated mice and increased secretory immunoglobulin A (sIgA) secretion and also repaired cyclophosphamide-induced mechanical barrier damage in the small intestine of mice, which could provide a basis for the development of anticancer drugs and chemotherapeutic agents. The kudzu polysaccharide ameliorates diarrhea in newborn calves by increasing the relative abundance of beneficial bacteria such as *Bifidobacterium* spp. and decreasing the relative abundance of pathogenic or diarrhea-associated bacteria such as *Escherichia* spp. and *Shigella* spp. (Shen et al., 2023). In addition, puerarin may improve the symptoms of abdominal pain and diarrhea in irritable bowel syndrome (IBS-D) rats by inhibiting the expression of corticotropin-releasing hormone receptor (CRF-1), which suppresses the activity of the hypothalamic–pituitary–adrenal (HPA) axis, promotes the proliferation of colonic epithelial cells by upregulating the expression of p-ERK/ERK, and repairs the colonic mucus by upregulating the expression of occludin, a protein that repairs the colonic mucus barrier (Wang Q.-S. et al., 2021). Puerarin also alleviates ulcerative colitis through the NF-κB/Nrf2 pathway (Jeon et al., 2020).

### 5.4 Respiratory system

PL modulates arginine metabolic pathways and attenuates bleomycin-induced pulmonary fibrosis symptoms in rats by decreasing nitric oxide synthase 2 (NOS 2)-related signaling molecules (Du et al., 2024). With the deepening of research, more and more results confirm that the components with respiratory system-promoting effects in PL are mainly isoflavonoid components. For example, puerarin can attenuate airway inflammation by inhibiting eotaxin-3 expression (Wang et al., 2015), ameliorate acute lung injury by downregulating NLRP3 (NOD-like receptor thermal protein domain-associated protein 3) inflammatory vesicle-induced pyroptosis (Cai D. et al., 2022), and have therapeutic effects on early chronic obstructive pulmonary disease (Zhang et al., 2022). It also ameliorates rabbit lung ischemia-reperfusion injury by inhibiting Fas/Factor-related apoptosis ligand (FasL) mRNA expression (Zheng et al., 2015). With the advancement of technology, kudzu-derived exosome-like nanovesicles were able to effectively attenuate inflammatory changes in the lungs by modulating the gut microbiota (Lu et al., 2024).

### 5.5 Urinary system

An aqueous extract of PL can ameliorate the overactivity of the forced urethral muscle in spontaneously hypertensive rats via a neurogenic pathway (Zhou et al., 2016). Combined with the existing studies, puerarin is one of the important active substances in PL with urinary effects. First, puerarin may bind to the Toll/interleukin 1 receptor (TIR) structural domain of the myeloid differentiation primary response protein 88 (MyD88) protein, impede its binding to Toll-like receptor 4 (TLR4), and downregulate NF-κB p65 and the c-Jun N-terminal kinase (JNK)/Forkhead box transcription factor O1 (FoxO1) pathway activity to attenuate lipopolysaccharide (LPS)- and unilateral ureteral obstruction (UUO)-induced renal inflammatory injury in C57BL/6J mice (Hu et al., 2024). Second, puerarin may upregulate renal Oat 1/3 by way of B-cell lymphoma 6 (BCL-6) upregulation of renal Oat 1/3 in methotrexate-induced kidney injury in Wistar rats (Liu et al., 2018). It is worth noting that some studies have confirmed that puerarin is the precursor component. In addition, a novel patch combining puerarin and polylactic acid using electrostatic spinning technology has good repair and pre-collagen synthesis properties for pelvic organ prolapse (Zhang D. et al., 2024), and it is expected to be an alternative to the traditional topical patch.

### 5.6 Reproductive system

As isoflavones with estrogen-like effects inhibit male spermatogenesis and female fertility to varying degrees (Sleiman et al., 2021), care should be taken with the dosage of the isoflavones of PL during physiologic states. It has been shown that the component of PL that inhibits sperm activity is not puerarin but other isoflavones (Gray et al. 2015).

### 5.7 Endocrine system

Extracts of PL have good anti-diabetic activity. For example, PL upregulates fatty acyl groups (FA), downregulates the levels of metabolites such as glycerophospholipids (GP) (Yang H.-B. et al., 2024), and interferes with the PI3K signaling pathway (Zhang et al., 2021) to ameliorate type II diabetes. In addition, kudzu extract may improve the development of diabetes by downregulating the PTP1B pathway (Sun R. et al., 2019), and kudzu polysaccharides may treat diabetes by modulating the PPAR signaling pathway and the structure of intestinal flora (Wang et al., 2022). It is worth mentioning that the effects of puerarin for the treatment of diabetes mellitus are manifested in various aspects such as lowering blood glucose levels, improving insulin resistance, and protecting pancreatic islets (Chen et al., 2019). As well, puerarin was able to promote pancreatic β-cell neogenesis by upregulating glucagon-like peptide 1 receptor (GLP-1R) expression, thereby ameliorating high-fat-diet-induced glycemic abnormalities in mice (Wang et al., 2020), and it also inhibited type II diabetic nephropathy by regulating iron homeostasis and attenuating iron death (Hou et al., 2024).

PL improves multiple female sexual dysfunctions in postmenopausal women by attenuating total cholesterol, triglycerides, alanine aminotransferase (ALT/GPT), and aspartate aminotransferase (AST/GOT) in ovariectomized rats (Oh et al., 2019). As for perimenopausal syndrome, osteoporosis is one of the most common disorders. An extract of PL can ameliorate postmenopausal osteoporosis by decreasing the levels of bone markers C-telopeptide of crosslinked collagen type I (CTX-1), osteocalcin (OC), and dye-decolorizing peroxidase (DYP) and upregulating ER-α protein expression in ovariectomized rats (Lee et al., 2021). Isoflavones from high-dose PL improved lipid and bone metabolism in ovariectomized mice by affecting elevated plasma estrogen levels, decreasing abdominal adipose tissue weight, and elevating femur BMD (Cho et al., 2012). Some studies have confirmed that puerarin has the effect of increasing localized new bone formation and can be used for bone grafting or osteoinduction (Wong and Rabie, 2007). In addition, puerarin promotes cell proliferation by upregulating Bcl-xL expression, promoting osteoblast proliferation and differentiated material through activation of MEK/ERK and PI3K/Akt pathways (Wang et al., 2013), and also inhibits osteoclastogenesis by reducing the pathway in which MCP-1 prevents migration of osteoclast precursor cells (OCPs) (Lin et al., 2020) and is expected to be a drug to prevent or delay osteoporosis. Wang et al. (2014) demonstrated that puerarin may exert anti-osteoporotic effects by inhibiting receptor activator of nuclear factor-κB ligand (RANKL) and interleukin-6 through increasing the expression of ERα, ERβ and steroid hormone receptor coactivator (SRC)-1. Subsequent studies have confirmed that puerarin inhibits osteoclastogenesis via autophagy response regardless of the presence of RANKL (Zhang G. et al., 2019), but the mechanism of autophagy in the anti-osteoporotic effect of puerarin has yet to be further explored. The combination of puerarin and zinc can be anti-osteoporotic by elevating serum osteocalcin, bone marrow stromal cell (BMSC) proliferation, and alkaline phosphatase (ALP) levels and decreasing serum lipocalin levels (Li H. et al., 2016). In addition to puerarin, other isoflavones in PL also have promising anti-osteoporotic effects, such as genistein and zinc that increase bone mass in de-ovulated rats (Qi, 2018) and puerarin 6″-O-Xyloside that is capable of anti-osteoporosis by elevating blood calcium, blood phosphorus, ALP, and OPG levels in a de-ovulated mouse model of anti-osteoporosis (Li H. et al., 2016).

Studies have shown that puerarin is able to alleviate Hashimoto’s thyroiditis by regulating macrophages (Tao et al., 2024a), which further confirms the favorable hormone-like effects of PL. Because PL contains estrogen-like isoflavones, it can also treat breast diseases.

### 5.8 Nervous system

High doses of PL isoflavonoids can promote the metabolism of dopamine (DA) and 5-hydroxytryptamine (5-HT), reduce the level of extracellular Glu, and have a good therapeutic effect on neurodegenerative diseases (Xiao et al., 2017), so the isoflavonoids of PL are one of the pharmacological material bases for the neurological activity of PL. First, PL has the most prominent therapeutic effect on Alzheimer’s disease. Puerarin forms a stable complex with calcium/calmodulin kinase IIα (CaMK IIα), a key protein for synaptic plasticity, which can alleviate the synaptic plasticity damage caused by Alzheimer’s disease through p38MAPK/cAMP-response element binding protein (CREB) signaling pathway (Liu S. et al., 2021) and inhibit the activity of acetylcholinesterase (AChE) in brain tissue. It also downregulated the expression of cyclooxygenase-2 (COX-2) and cysteine-3 (C3) to exert anti-Alzheimer’s disease effects (Liu et al., 2019) and decreased cognitive deficits in lead acetate-induced neurotoxicity mice by elevating the activities of acetylcholinesterase (AChE) and monoamine oxidase (MAO) and modulating the PKA/Akt/NOS signaling pathway (Liu et al., 2013). For Aβ1-42-induced cognitive deficits and neurodegenerative lesions, puerarin also has a favorable protective effect, mainly by elevating the levels of brain-derived neurotrophic factor, decreasing AChE, *etc.*, in the hippocampus and cerebral cortex (Wu et al., 2017). Furthermore, the lupane triterpenoids isolated from PL can attenuate Alzheimer’s disease by inhibiting beta-site APP cleaving enzyme 1 (BACE1) (Koirala et al., 2017). Not to be overlooked, PL and its active components also inhibit hypoxic brain edema (Wang C. et al., 2018). It also attenuates ischemic stroke injury by modulating intestinal flora (Lian et al., 2023) and is anti-stroke by inhibiting lactate dehydrogenase (Li et al., 2017).

### 5.9 Immune system

PL has anticancer and immunity-enhancing effects on the immune system. First, isoflavones (biochanin A and genistein) of PL with 5,7-dihydroxy groups on the A-ring in their structure can exert anticancer effects by inhibiting NO production (Jun et al., 2005). Puerarin’s anticancer effects may be carried out through PI3K/AKT/mammalian target of rapamycin (mTOR), Ras-Raf-MEK-ERK, JNK, AMPK, and NF-κB pathways (Ahmad et al., 2020). Second, studies on the immunity-enhancing effects of PL mainly include the ability of polysaccharides of PL to inhibit cyclophosphamide-induced immunocompromise by increasing immune organ index, cytokine content, and T-lymphocyte subsets (Cai et al., 2023).

### 5.10 Other activities

**FIGURE 10:**
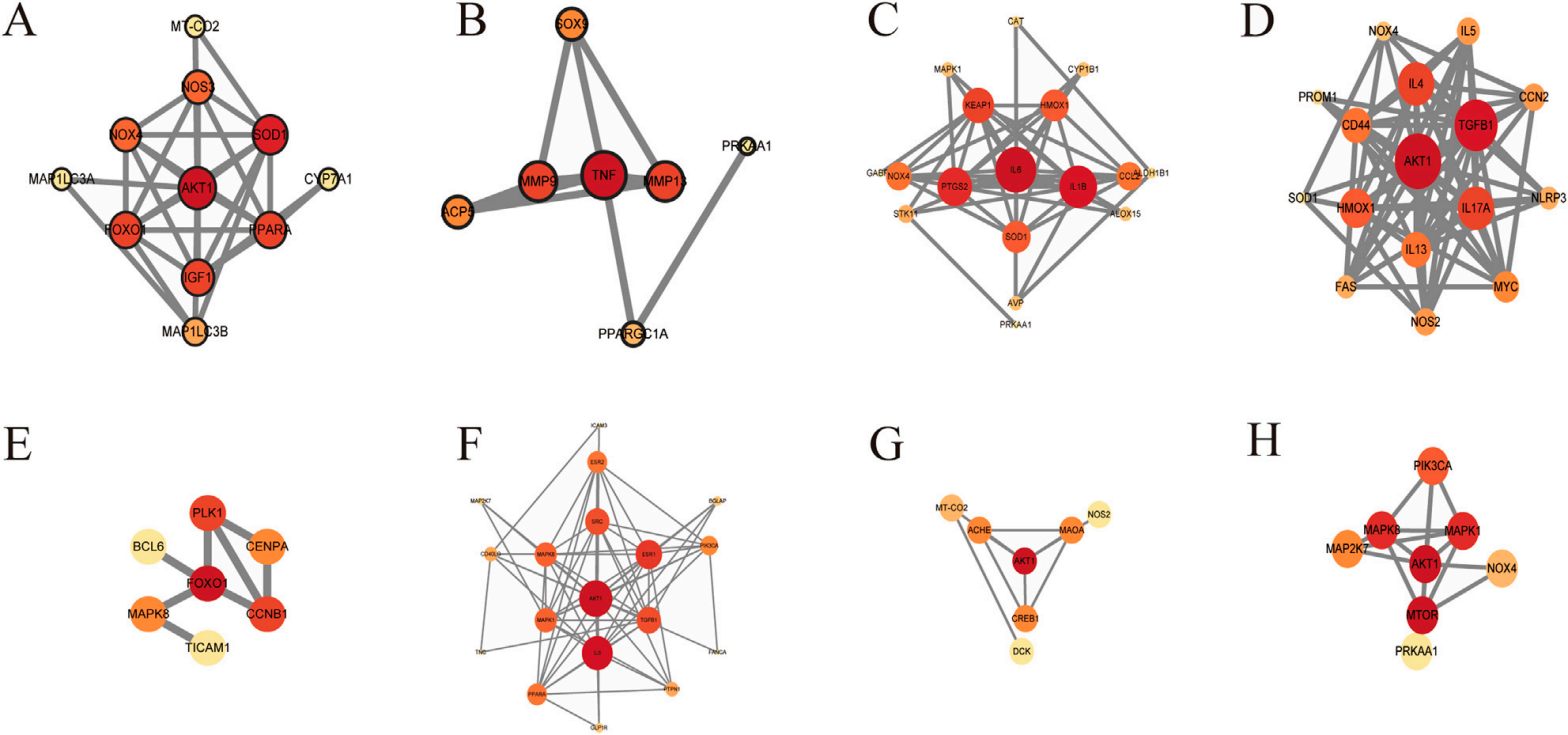
PPI network diagrams of targets in the **(A)** circulatory, **(B)** locomotor, **(C)** digestive, **(D)** respiratory, **(E)** urinary, **(F)** endocrine, **(G)** nervous, and **(H)** immune systems.

With the help of the STRING database (https://string-db.org/) and Cytoscape 3.8.2 software (https://cytoscape.org/download.html), the targets described above were visualized (Figure 10). In addition to the role of the reproductive system, the results of the PPI network diagram visualization of the other eight systems showed that AKT, IL-6, TGF-β, AMPK, SOD, TNF, and MMP were the core targets for PL to play the above roles. In addition, PL also has the effects of skin barrier damage protection (Lee et al., 2024), anti-fatigue (Xiaoming et al., 2012), suggesting it could be added to cosmetics as a whitening agent or to food as an anti-fatigue agent, *etc.*. It has been shown to have a good safety profile in normal doses (Chen et al., 2021), with no obvious toxic side effects, and it has great potential for food development.

In summary, the ancient usage of PL has certain modern scientific connotations, such as the ability to detoxify muscles and reduce fever, corresponding to anti-inflammation, and its mechanism mainly includes inhibiting COX-2 enzyme activity, decreasing PGE2 synthesis, and decreasing the expression of pro-inflammatory factors, such as TNF-alpha and IL-6. In addition, ancient books report that PL can treat thirst and relieve alcoholism, which corresponds to the modern studies showing that it can regulate insulin resistance and treat alcoholic liver injury, respectively. However, the mechanisms remain to be further elucidated, and subsequent studies on PL should focus on the above points.

## 6 Food utilization

### 6.1 Preservative

The key enzyme in this process is polyphenol oxidase, which is responsible for the reduction of the nutritional value of PL due to enzymatic browning during storage and processing. Liu J. et al. (2021) showed that the polyphenol oxidase of PL has a high thermal stability, and that the food components pectin, bovine serum proteins, starch, and sucrose showed a decreasing trend in their protective effect against thermal processing. This further suggests that the addition of sucrose and starch during processing or storage is effective in reducing enzymatic browning and enhancing the quality of PL. Researchers added a probiotic solution to the herbal combination consisting of chuanxiong, licorice, forsythia, safflower, kudzu, and galangal, and fermented it at 30°C and pH 6.5 for 8 days. The effect on citrus fruit decay was later tested, and it was found that the herbal fermentation solution was able to inhibit decay significantly. It is a novel, highly effective, and natural citrus freshness preservative (Wang et al., 2019a).

### 6.2 Tea

Researchers used PL as the main ingredient and added herbs such as maitake, *Ganoderma lucidum*, and American ginseng, which are functional foods and medicines, to make *Ge Gen Mai Dong Cha*, a health food that has the potential to lower glucose and combat obesity (Li X. et al., 2023). The extracts of PL tea have good preservative and bacteriostatic effects and can effectively inhibit the growth of Gram-negative and positive pathogenic bacteria in various food products (Kim and Fung, 2004). Koreans use kudzu as a non-alcoholic beverage by soaking it in water or adding sweeteners such as honey and sugar, called yakjaecha (Lee, 2022).

### 6.3 Fermented products

Wang Y. et al. (2023) found that the mixed fermentation of *A. erythropolis* with rice, PL, and yam resulted in the conversion of soybean glycosides and diosgenin to daidzein and diosgenin, respectively, which were more efficiently digested and absorbed *in vivo*.

### 6.4 Kudzu starch with food activity

Kudzu starch is a powdered substance extracted from dried tubers of PL after water milling that has been shown to have circulation-enhancing and antispasmodic properties (Mocan et al., 2018). Robert D. Tanner et al. devised a lysine-enriched baker’s yeast process and found that kudzu starch was similar to commercially available fermented starches (Tanner and Hussain, 1979). Due to the rich nutrient content of kudzu starch, it is used as a drinkable solid beverage in Asia (Liang et al., 2017). Researchers have studied the properties of three kudzu starches (Duan et al., 2023) that can be used as an ingredient for cookies based on the properties. He et al. (2024) improved the kudzu starch extraction method and obtained kudzu starch that could be used as a binder and thickener after treatment with an alcohol base and a urea base. Because kudzu starch is rich in nutrients, it is often developed as a functional food with health benefits in East Asian countries such as China and Japan (Van Hung and Morita, 2007; Jóźwiak et al., 2018).

### 6.5 Others

In India, PL is commonly used as an additive in dairy products and may help regulate blood pressure by stimulating the body’s physiological systems (Maji et al., 2023). Yu et al. (2017) constructed a large-scale knowledge graph to demonstrate the nutritional value of PL, using Ge Gen Fen Zhou as an example.

iu-Zhi Fan et al. found that the use of substrates of mulberry branches and PL residues for the cultivation of monkey head mushrooms, on the one hand, could improve the yield and biological efficiency; on the other hand, the mushrooms harvested from the alternative substrates were rich in flavonoids, polysaccharides, and other active constituents, which significantly improved their antioxidant and hypoglycemic activities and enhanced the value of monkey head mushrooms (Fan et al., 2021).

Corley et al. (1997), using the above-ground parts (leaves and stems) and roots of PL, found that the chemical composition and digestive properties of these parts were very close to those of other commonly used feedstuffs, showing its potential as a feed for ruminants. In addition, the addition of PL leaves to feeds significantly increased broiler growth performance, immunity, and antioxidant capacity (Gong et al., 2022).

## 7 Toxicity

It can be concluded from the above ancient texts that kudzu is not recorded as poisonous in most of the *Materia Medica* texts, but rather that most of the texts mention its edible properties. Song et al. (2020) found that the LD_50_ (lowest dose causing death) of oral PL extract was greater than 5,000 mg/kg, and no acute toxicity was observed. Although no significant toxicity has been observed with oral administration of extracts of PL, injections of high concentrations of *Pueraria mirifica* have been reported in the literature to cause hemolysis (0.2–1 mM without significant side effects) (Hou et al., 2011). In addition, high concentrations (>1 mg/kg) of puerarin had deleterious effects *in vitro* and *in vivo* on oocyte maturation, fertilization, and subsequent embryonic development in mice (Huang and Chan, 2016). Therefore, the dosage of the medicinal components of *Pueraria mirifica* should be controlled in the actual application process to ensure that no toxicity or side effects will occur.

## 8 Discussion

### 8.1 Industrialization challenges in the medicinal–edible process

The thermal stability of *Pueraria* isoflavones (notably puerarin and daidzein) constitutes a critical challenge in industrial processing, as these bioactive compounds exhibit susceptibility to thermal degradation under conventional thermal treatments. Such thermal degradation directly compromises the bioactivity of *Pueraria* extracts, necessitating the development of thermal-protective strategies during processing to preserve critical components like heat-sensitive isoflavones (e.g., genistein). Recent work by Ekumah et al. (2025) demonstrated that a kudzu starch hydrogel functionalized with rutin and ferulic acid (RF-KSH) reduces component loss by 18%–22% while increasing antioxidant activity by 1.3-fold compared to conventional hydrogels. The system was further optimized through octenyl succinic anhydride modification, specifically designed to enhance thermal stability during processing.

Conventional ethanol-based extraction methods are constrained by a fundamental compromise between polysaccharide retention and industrial costs. Higher ethanol concentrations not only induce polysaccharide precipitation but also lead to significantly increased solvent recovery costs, creating dual challenges for process optimization. To address these limitations, researchers are developing innovative extraction strategies. Emerging green techniques such as ultrasound-assisted and microwave-assisted extraction demonstrate dual benefits: enhanced recovery of polysaccharides and isoflavones coupled with reduced environmental impact, while simultaneously improving cost efficiency through optimized energy consumption (Xu et al., 2025). The industrial utilization of PL requires systematic optimization to fully realize its strategic potential in functional foods and pharmaceuticals; this demands coordinated efforts in both fundamental research and applied technological development to effectively harness its medicinal-food duality.

### 8.2 Future research directions

Studies have confirmed the anti-inflammatory and immunomodulatory properties of *Pueraria* isoflavones (e.g., puerarin and daidzein). However, critical knowledge gaps remain regarding signaling pathway interactions between their microbial metabolites (e.g., equol) and specific immune cell populations (macrophages and T lymphocytes). Key unresolved issues encompass 1) gut microbiota-mediated modulation of isoflavone metabolic pathways during immunoregulation and 2) epigenetic regulation through DNA methylation/histone modifications induced by isoflavones. Both require systematic investigation *via* integrated application of single-cell sequencing and metabolomics. While the hypoglycemic properties of *Pueraria* polysaccharide have been extensively studied, the relationship between its structural parameters (particularly branching degree and glycosidic linkage types) and immunomodulatory potential remains unelucidated. To elucidate the stereocomplementary interaction mechanisms between polysaccharides and immune receptors, structural biology approaches, including molecular dynamics simulations and ligand–receptor binding assays, should be systematically implemented.

*Pueraria* isoflavones face significant application constraints due to poor aqueous solubility and low intestinal absorption rates. Current strategies employing liposome encapsulation technology and pH-responsive release systems demonstrate bioavailability enhancement potential. However, nanodrug delivery systems with therapeutic payloads remain at experimental stages. Critical challenges persist regarding nanomaterial accumulation-related chronic toxicity in hepatic/renal tissues and the development of scalable manufacturing processes.

## 9 Conclusion

In this article, the research progress of PL is systematically summarized from the aspects of basal origin, chemical composition, synthesis, metabolism, biological activity, and comprehensive utilization of food. This is of great significance for the further development and application of PL. PL is mainly distributed in Asia, America, and Europe and has been used as a medicinal herb and food for thousands of years in Asia (especially in China), while the utilization value of *Pueraria lobata* in Europe and the United States has yet to be further developed. PL mainly contains isoflavonoids, and the methylation and glycosylation of 6-C, 8-C, 3′-C, 4′-C are its main biogenic pathways. Hepatic and intestinal metabolism, glucuronidation, methylation, reduction, and oxidation reactions are the main types of reactions. Resorcinol, (3S)-equol, and equol are its metabolic end products, and puerarin, daidzein, genistein, biochanin A, and formononetin are the key components affecting its quality and activity.

**FIGURE 11:**
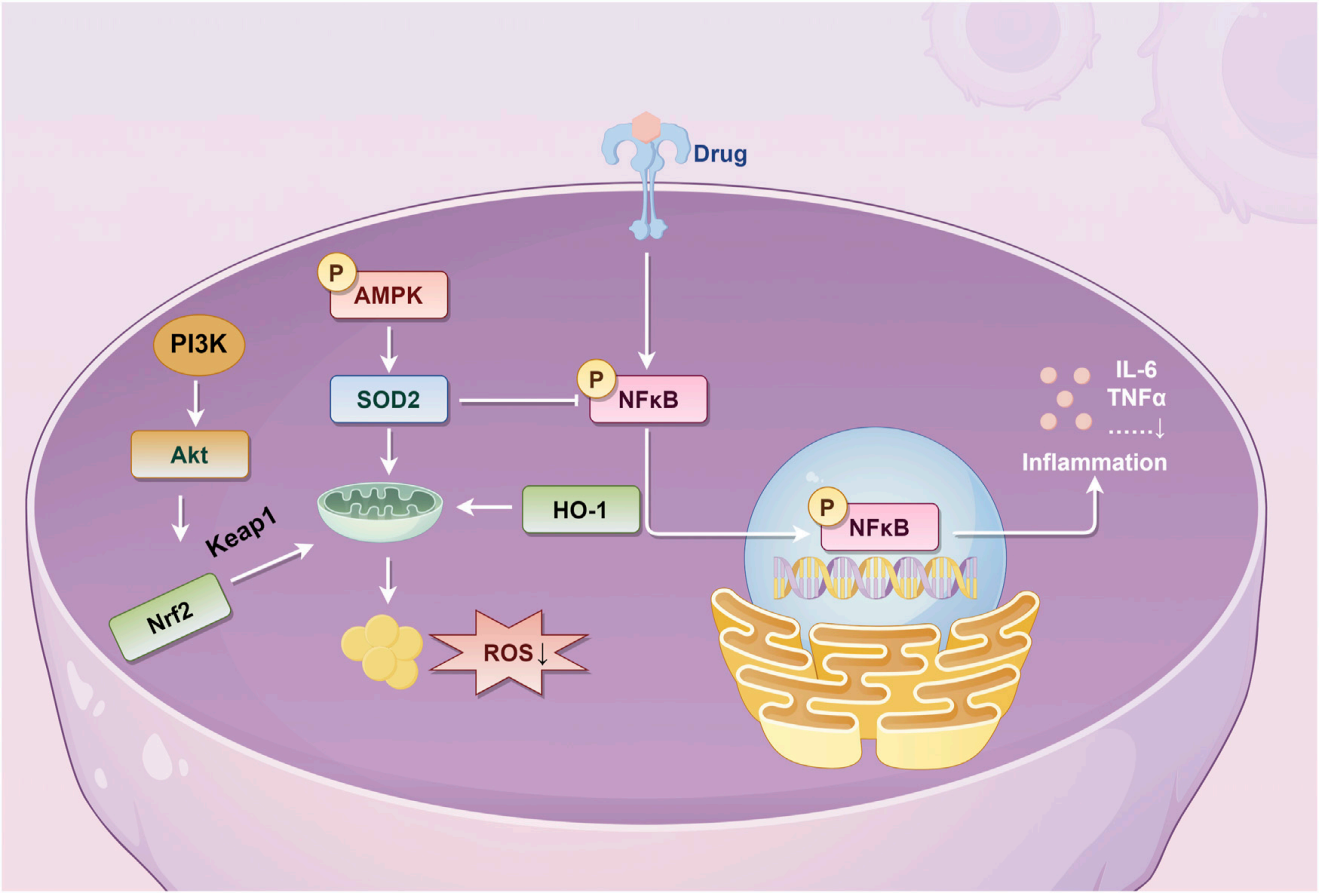
Mechanistic diagram of PL’s effects in cells.

As a natural herb with both medicinal and functional properties, PL exhibits three pivotal advantages for modern applications. First, its multi-target regulatory capacity extends beyond modulating key signaling molecules (e.g., AKT, IL-6, and TGF-β) to bidirectional coordination of the Nrf2/AMPK pathways, a critical mechanism for achieving therapeutic-nutritional synergy (Figure 11). Second, PL demonstrates exceptional food matrix compatibility through heat-resistant starch that stabilizes bioactive isoflavonoids during thermal processing, enabling functional food development without compromising bioactivity. Third, its ecological sustainability is evidenced by significantly higher biomass utilization efficiency than conventional medicinal plants, which facilitates green manufacturing through full-plant utilization and byproduct valorization. These attributes collectively position PL as a strategic candidate for developing next-generation nutraceuticals and sustainable bioproducts.

PL can be used not only as food, but also as a tea, a preservative, and as an ingredient for making porridge, dairy products, food additives, cookies, jellies, puddings, and so on. Kudzu has a wide range of biological activities on the nine systems of the human body and can be used as nutritional enhancers and supplements. The main role is focused on liver function protection, anti-osteoporosis, and anti-diabetes, AKT, IL-6, TGF-β, AMPK, SOD, TNF, MMP, *etc.*, for the PL to play the role of the above core targets. Building on these strengths, two industrial implementation strategies emerge: 1) Development of gut microbiota-personalized nutraceuticals leveraging PL’s ability to modulate equol-producing bacterial communities and 2) advanced 3D food printing technologies for creating bone-targeted functional matrices that combine puerarin’s bioactivity with structural biomimicry. These approaches collectively position PL as a next-generation platform for precision nutrition and sustainable bioproduct development.

## Glossary

4CL: 4-Coumarate-CoA ligase
5-HT: 5-Hydroxytryptamine
ACC: Acetyl-CoA carboxylase
AChE: Acetylcholinesterase
ADH: Alcohol dehydrogenase
ADT: Plant aronic acid dehydratase
Akt: Protein kinase B
ALD: Alcoholic liver disease
ALDH: Acetaldehyde dehydrogenase
ALOX15: Arachidonic acid 15-lipoxygenase
ALP: Alkaline phosphatase
ALT: Alanine aminotransferase
ALT/GPT: Alanine aminotransferase
AMPK: Adenosine 5′-monophosphate (AMP)-activated protein kinase
APTT: Activated partial thromboplastin time
AST: Aspartate transaminase
AST/GOT: Aspartate aminotransferase
BACE1: Beta-site APP cleaving enzyme 1
BCL 6: B-cell lymphoma 6
BMD: Bone mineral density
BMSCs: Bone marrow mesenchymal stem cells
C3: Cysteine-3
C4H: Cinnamate 4-hydroxylase
CaMK IIα: Calcium/calmodulin kinase IIα
CAT: Catalase
CCNB 1: Cell cycle protein B1
CHI: Chalcone isomerase
CHR: Chalcone reductase
CHS: Chalcone synthase
COL10A 1: Collagen type X alpha 1 chain
COL2A 1: Collagen type II alpha 1 chain
COX-2: Cyclooxygenase-2
CREB: cAMP-response element binding
CRF: Corticotropin-releasing hormone receptor
CTGF: Connective tissue growth factor
CTX-1: C-telopeptide of crosslinked collagen type I
CWI: Cell-wall integrity
CYP1B1: Cytochrome P450 family 1 subfamily B member 1
CYP7A1: Cholesterol 7 alpha-hydroxylase
DA: Dopamine
DgpA: GenBank: BBG22493.1
DgpBC: PDBID: EM Map EMD-30808
DYP: Dye-decolorizing peroxidase
DZE: 0.30 0.02 O. D
eNOS: Endothelial nitric oxide synthase
ERK: Extracellular signal-regulated kinase
FA: Fatty acyl groups
FasL: Factor-related apoptosis ligand
FoxO1: Forkhead box transcription factor O1
GLP-1R: Glucagon-like peptide 1 receptor
GP: Glycerophospholipids
GR: Glutathione reductase
GSH: Glutathione
HID: 2-Hydroxyisoflavanone dehydratase
HO-1: Heme oxygenase-1
HPA: Hypothalamic–pituitary–adrenal
IBS-D: Irritable bowel syndrome
IGE: Immunoglobulin E
IGF: Insulin-like growth factor
IgM: Immunoglobulin
IL: Interleukin
JNK: c-Jun N-terminal kinase
Keap1: Kelch-like ECH-associated protein 1
LKB1: Liver kinase B1
LPS: Lipopolysaccharide
LTC4: Leukotriene C4
M3: Muscarinic receptor-3
MAO: Monoamine oxidase
MCP: Monocyte chemotactic protein
MDA: Malondialdehyde
MMP: Matrix metalloproteinase
MMP-9: Matrix metalloprotein-9
mTOR: Mammalian target of rapamycin.
MyD88: Myeloid differentiation primary response protein 88
NF-κB: Nuclear factor kappa-B
NLRP3: NOD-like receptor thermal protein domain-associated protein 3
NOS 2: Nitric oxide synthase 2
Nrf2: Nuclear respiratory factor 2
OC: Osteocalcin
OCPs: Osteoclast precursor cells
PAL: Phenylalanine ammonia-lyase
PGC-1: Peroxisome proliferator-activated receptor-γ coactivator
PGE 2: Prostaglandin E2
PGI2: Prostaglandin-I-2
phAT: Plant phosphotransacetylase
PI3K: Phosphatidylinositol 3-kinase
PL: *Pueraria lobata*
PLK 1: Polo-like kinase 1
PPAR: Peroxisome proliferator-activated receptors
PTGS2: Prostaglandin-endoperoxide synthase 2
PUE: 1.96 0.09 optical density (O.D.)
RANKL: Receptor activator of nuclear factor-κB ligand
sIgA: Secretory immunoglobulin A
SOD: Superoxide dismutase
SOX: SRY-related high-mobility-group box
SRC: Steroid hormone receptor coactivator
TC: Total cholesterol
TG: Triglycerides
TGF-β: Transforming growth factor-beta
Th 17: T helper cell 17
Th 2: T helper 2 cell
TIR: Toll/interleukin 1 receptor
TLR4: Toll-like receptor 4
TNF: Tumor necrosis factor
TRAP: Tartrate acid phosphatase
TT: Thrombin time
TXA2: Thromboxane A2
UUO: Unilateral ureteral obstruction
